# Comparative cardiovascular benefits of individual SGLT2 inhibitors in type 2 diabetes and heart failure: a systematic review and network meta-analysis of randomized controlled trials

**DOI:** 10.3389/fendo.2023.1216160

**Published:** 2023-12-20

**Authors:** Tanawan Kongmalai, Phorntida Hadnorntun, Pattara Leelahavarong, Pinkawas Kongmalai, Varalak Srinonprasert, Srisakul Chirakarnjanakorn, Usa Chaikledkaew, Gareth McKay, John Attia, Ammarin Thakkinstian

**Affiliations:** ^1^ Mahidol University Health Technology Assessment (MUHTA) Graduate Program, Mahidol University, Bangkok, Thailand; ^2^ Siriraj Health Policy Unit, Faculty of Medicine, Siriraj Hospital, Mahidol University, Bangkok, Thailand; ^3^ Division of Endocrinology and Metabolism, Department of Medicine, Faculty of Medicine, Siriraj Hospital, Mahidol University, Bangkok, Thailand; ^4^ Department of Orthopedics, Faculty of Medicine, Kasetsart University, Bangkok, Thailand; ^5^ Division of Geriatric Medicine, Department of Medicine, Faculty of Medicine, Siriraj Hospital, Mahidol University, Bangkok, Thailand; ^6^ Division of Cardiology, Department of Medicine, Faculty of Medicine, Siriraj Hospital, Mahidol University, Bangkok, Thailand; ^7^ Department of Pharmacy, Faculty of Pharmacy, Mahidol University, Bangkok, Thailand; ^8^ Centre for Public Health, School of Medicine, Dentistry, and Biomedical Sciences, Queen’s University, Belfast, United Kingdom; ^9^ School of Medicine and Public Health, University of Newcastle, Callaghan, NSW, Australia; ^10^ Department of Clinical Epidemiology and Biostatistics, Faculty of Medicine, Ramathibodi Hospital, Mahidol University, Bangkok, Thailand

**Keywords:** sodium-glucose cotransporter 2 inhibitor (SGLT2 inhibitor), congestive heart failure, cardiovascular disease, diabetes mellitus, systematic review, network meta-analysis

## Abstract

**Background:**

In patients with type 2 diabetes (T2D) and a history of heart failure (HF), sodium–glucose cotransporter-2 inhibitors (SGLT2is) have demonstrated cardiovascular (CV) benefits. However, the comparative efficacy of individual SGLT2is remains uncertain. This network meta-analysis (NMA) compared the efficacy and safety of five SGLT2is (canagliflozin, dapagliflozin, empagliflozin, ertugliflozin, and sotagliflozin) on CV outcomes in these patients.

**Materials and methods:**

PubMed, Embase, and the Cochrane Central Register of Controlled Trials were searched up to September 23, 2022, to identify all randomized controlled trials (RCTs) comparing SGLT2is to placebo in T2D patients with HF. The main outcomes included composite CV death/heart failure hospitalization (HFH), HFH, CV death, all-cause mortality, and adverse events. Pairwise and NMA approaches were applied.

**Results:**

Our analysis included 11 RCTs with a total of 20,438 patients with T2D and HF. All SGLT2is significantly reduced HFH compared to standard of care (SoC) alone. “Add-on” SGLT2is, except ertugliflozin, significantly reduced composite CV death/HFH relative to SoC alone. Moreover, canagliflozin had lower composite CV death/HFH compared to dapagliflozin. Based on the surface under the cumulative ranking curve (SUCRA), the top-ranked SGLT2is for reducing HFH were canagliflozin (95.5%), sotagliflozin (66.0%), and empagliflozin (57.2%). Head-to-head comparisons found no significant differences between individual SGLT2is in reducing CV death. “Add-on” SGLT2is reduced all-cause mortality compared with SoC alone, although only dapagliflozin was statistically significant. No SGLT2is were significantly associated with serious adverse events. A sensitivity analysis focusing on HF-specific trials found that dapagliflozin, empagliflozin, and sotagliflozin significantly reduced composite CV death/HFH, consistent with the main analysis. However, no significant differences were identified from their head-to-head comparisons in the NMA. The SUCRA indicated that sotagliflozin had the highest probability of reducing composite CV death/HFH (97.6%), followed by empagliflozin (58.4%) and dapagliflozin (44.0%).

**Conclusion:**

SGLT2is significantly reduce the composite CV death/HFH outcome. Among them, canagliflozin may be considered the preferred treatment for patients with diabetes and a history of heart failure, but it may also be associated with an increased risk of any adverse events compared to other SGLT2is. However, a sensitivity analysis focusing on HF-specific trials identified sotagliflozin as the most likely agent to reduce CV death/HFH, followed by empagliflozin and dapagliflozin.

**Systematic review registration:**

https://www.crd.york.ac.uk/prospero/, identifier CRD42022353754.

## 1 Introduction

Heart failure (HF) is a prevalent and debilitating complication of type 2 diabetes (T2D), contributing to increase morbidity and mortality in affected individuals. Worldwide, more than 26 million people are affected by this condition (1, 2), and T2D is a well-established risk factor with approximately 10%–30% of T2D patients aged over 70 years reported to have had HF (3).

Comorbid T2D with cardiovascular disease (CVD) is associated with higher mortality (4), highlighting the importance of reducing the risk of CVD in T2D management. A previous systematic review and meta-analysis (SRMA) indicated that intensive glucose lowering was not significantly associated with CVD risk reduction but conversely increased HF by 47% (5). Novel strategies are therefore necessary to improve prognosis and lower mortality in patients with T2D.

Sodium–glucose cotransporter-2 inhibitors (SGLT2is) are relatively recent oral anti-diabetic drugs (OADs) that decrease renal tubular glucose reabsorption (6). Although they provide modest glycemic control, cardiometabolic and hemodynamic improvements are evident with a low risk of hypoglycemia (7, 8). As a result, SGLT2is are strongly recommended in clinical practice guidelines for HF (9, 10). Although individual SGLT2is have similar mechanistic effects, pharmacological variations have resulted in variable efficacy and safety in cardiovascular outcome trials (CVOTs) (11, 12), making the most appropriate choice of SGLT2i challenging. For instance, sotagliflozin has the lowest SGLT2/SGLT1 selectivity, canagliflozin has the lowest oral bioavailability, empagliflozin has the highest SGLT2 protein selectivity, and ertugliflozin has the highest oral bioavailability (13). Although several studies have shown the benefits of SGLT2is in reducing heart failure hospitalization (HFH) in T2D (14–19), the effects reported for each SGLT2i vary, and the effects on cardiovascular (CV) and all-cause mortality were inconsistent (14–21).

The associated costs of SGLT2is also limit their accessibility, especially in limited-resource settings. The cost for SGLT2i therapy in the United States ranged from $405.98 to $426.27/person/month, with an out-of-pocket cost of $36.76 to $56.64/person/month (22). However, given the variable individual medication pricing, an improved understanding of individual SGLT2i efficacy and safety will inform treatment decisions. Direct head-to-head comparisons of all SGLT2is are unlikely; a network meta-analysis (NMA) may provide indirect comparisons and a ranking of the efficacy and safety of individual SGLT2is.

Although several trials were conducted in patients with T2D, only ~10% had previously reported HF (19, 23, 24), in contrast to the ~50% of HF patients who had previously reported T2D (14, 15, 17, 20, 25). None of these studies were sufficiently powered to evaluate SGLT2i efficacy in HF-T2D patients. Previous SRMAs have evaluated the efficacy of SGLT2is in various patient groups (26–29), although none have specifically targeted HF-T2D or used an NMA approach. Therefore, this NMA was conducted to compare the CV benefits and adverse events (AEs) associated with individual SGLT2is in HF-T2D patients. Specifically, we aimed to determine which SGLT2i provides the greatest efficacy in reducing cardiovascular events in this patient population.

## 2 Materials and methods

This study followed the Preferred Reporting Items for Systematic Reviews and Meta-Analyses (PRISMA) statement (30) and was registered in PROSPERO (CRD42022353754).

### 2.1 Data sources, search strategy, and data extraction

Three electronic databases, PubMed, Embase, and the Cochrane Central Register of Controlled Trials, were searched from inception to August 15, 2022, with an update on September 23, 2022, without language restrictions. The search terms and strategies are provided in **Supplementary Table S1**. The titles and abstracts were reviewed by two independent reviewers (TK and PH), and disagreements were resolved with a third reviewer (PL). The inclusion criteria included i) randomized controlled trials (RCTs) or their subgroup or *post-hoc* analyses of SGLT2is in HF-T2D, ii) compared SGLT2is with standard of care (SoC), and iii) included any outcome of interest (i.e., composite CV death/HFH, HFH, CV death, and all-cause mortality).

Two reviewers (TK and PH) independently extracted the data; disagreements were adjudicated by PL. The data extractions included i) study characteristics (i.e., study participants and number, study design, follow-up period, age, sex, baseline ejection fraction (EF), HF type (preserved/reduced EF), functional class (New York Heart Association (NYHA) Functional Classification), and other concomitant medications); (ii) interventions (SGLT2i type, dose, and duration); and (iii) outcomes (i.e., composite CV death/HFH, HFH, CV death, all-cause mortality, and AEs).

### 2.2 Interventions, comparator, and outcomes of interest

Interventions included individual SGLT2is, i.e., dapagliflozin (5 and 10 mg), canagliflozin (100 and 300 mg), empagliflozin (10 and 25 mg), sotagliflozin (200 and 400 mg), and ertugliflozin (5 and 15 mg).

Comparators included placebo or SoC for HF. HF treatment included device therapies, such as implantable cardioverter defibrillators and cardiac resynchronization therapy if indicated, in addition to medications, including diuretics, beta-blockers, mineralocorticoid receptor antagonists (MRAs), angiotensin-converting enzyme inhibitors (ACEIs), angiotensin receptor blockers (ARBs), and sacubitril–valsartan.

The primary outcome included composite CV death/HFH originally defined by individual RCTs. Secondary outcomes included HFH, CV death, all-cause mortality, and any AEs (i.e., volume depletion, acute kidney injury (AKI), urinary tract infection, genital tract infection, diabetic ketoacidosis (DKA), and bone fracture) in addition to serious AEs (SAEs). SAEs were defined as i) death or immediate life-threatening event, ii) persistent or clinically significant disability or incapacity, iii) events requiring hospitalization, iv) events related to a congenital anomaly or birth defect, or v) deemed serious for any other reason (14, 31, 32).

### 2.3 Risk of bias assessment

Two authors (TK and PK) independently assessed the risk of bias (RoB) using the Cochrane Risk of Bias tool version 2 (RoB2) based on five domains: randomization process, deviations from the intended protocol, missing outcome data, measurement of the outcomes, and selection of the reported result. Disagreements were adjudicated by PL. The overall quality was graded as high, with some concern and a low risk of bias (33).

### 2.4 Statistical analysis

Effect sizes (i.e., unstandardized mean difference (USMD) and risk ratio (RR)) along with 95% confidence intervals (CIs) were estimated for continuous data and dichotomous outcomes. Heterogeneity was assessed using the Q-test and I^2^ statistics. If heterogeneity was present (Q test <0.1 or I^2^ > 50% (34)), a meta-analysis (MA) random-effects model was used; otherwise, a fixed-effects model was considered. A meta-regression investigated the heterogeneity source by fitting each co-variable in the model including age, sex, baseline EF, HF type (reduced or preserved EF), functional class (NYHA), SoC with any HF treatment (i.e., MRA, renin–angiotensin system inhibitor [RASi], and angiotensin receptor/neprilysin inhibitor [ARNI]), treatment duration, and acute/chronic HF.

A two-stage NMA was applied as follows. First, a relative treatment effect (i.e., lnRR and USMD) was estimated with common variance–covariance. Second, treatment effects were pooled across studies using a multivariate MA with a consistency model. Transitivity was evaluated by exploring patient characteristics between comparisons or intervention arms, where appropriate. The inconsistency assumption was assessed using a global design-by-treatment interaction model, if applicable. Relative treatment effects were ranked using a rankogram and surface under the cumulative ranking curve (SUCRA). Publication bias was assessed using Egger’s test and adjusted comparison funnel plots, which, if asymmetrical, were evaluated further using a contour-enhanced funnel plot.

Subgroup/sensitivity analyses were pre-planned by HF type (preserved and reduced EF) and concomitant use of ARNI, MRA, and RAS blockade as SoC, if data were available. Furthermore, clustered-ranking plots were used to evaluate and rank risks and benefits associated with individual SGLT2is.

STATA version 17 (StataCorp, College Station, TX, USA) was used for all analyses. A significance threshold of p < 0.05 was considered, except for the heterogeneity and Egger’s tests, where a p-value <0.10 was used.

## 3 Results

**Figure 1:**
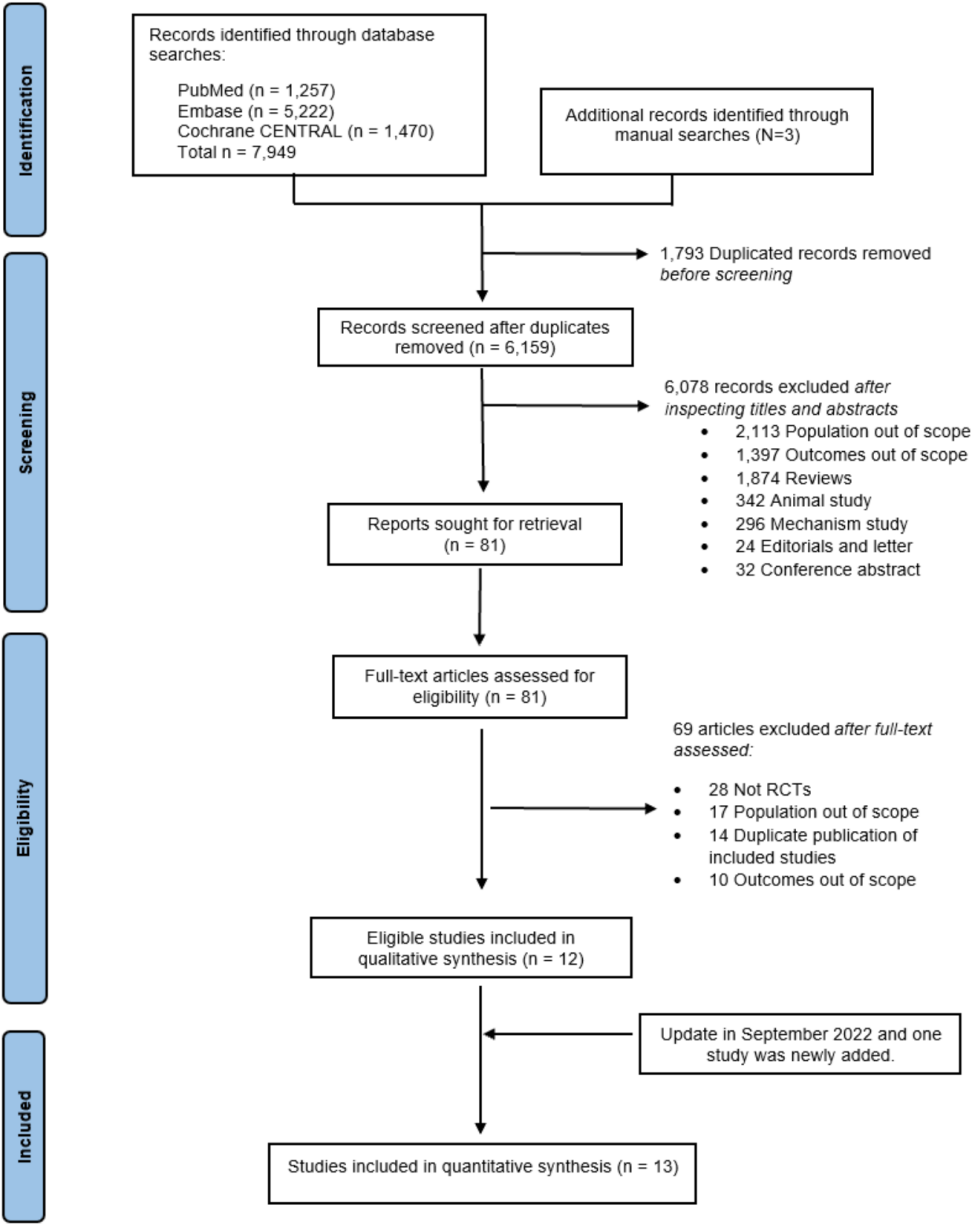
PRISMA flow diagram. PRISMA, Preferred Reporting Items for Systematic Reviews and Meta-Analyses.

A total of 7,952 articles were identified, but only 12 met the eligibility criteria. An updated search conducted on September 23, 2022, revealed one additional study (25). Among the 13 publications, two (14, 35) were from SOLOIST-WHF, and two were from EMPA-REG (31, 36), each reporting different outcomes of interest. This resulted in 13 articles drawn from 11 RCTs for inclusion within this NMA (see **Figure 1**). Among these, four RCTs assessed SGLT2is in patients with HF, with or without T2D (DAPA-HF (32), EMPEROR-reduced (37), EMPEROR-preserved (20), and DELIVER (25)), and two RCTs originally recruited patients with HF and T2D at baseline (CANONICAL (38) and SOLOIST-WHF (14, 35), hereafter referred to as “HF-specific” trials), while five RCTs (EMPA-REG outcome (31, 36), CANVAS (24), DECLARE-TIMI 58 (39), VERTIS-CV (40), and SCORED (41)) reported HF outcomes in subgroup or *post-hoc* analyses in T2D patients, hereafter called “T2D-specific” trials.

Study characteristics are summarized in **Table 1**; the median age was 67.3 years, and the percentage of female was 2.3 to 43.9, with a median follow-up time of 0.5 to 3.6 years (mean 1.7 years). There was evidence of elevated N-terminal prohormone of B-type natriuretic peptide (NT-proBNP) in all HF-specific trials, but no reports in T2D-specific trials. NYHA was mainly class II. The HF medications at baseline are shown in **Supplementary Table** **S2**.

**Table 1:** Characteristics of studies included in the quantitative analysis.

Study, year	N	Intervention	Dose (mg/day)	Follow-up (years)	Age (years)	Female, %	Duration of DM (years)	HbA1C (%)	BMI (kg/m^2^)	LVEF (%)	NT-proBNP (pg/ml)	eGFR (ml/min/1.73 m^2^)	NYHA II	NYHA III	NYHA IV
EMPA-REG, 2016 (31, 36)	706	Empagliflozin	10, 25	3.1	64.50	29.90	NA	8.08	32.04	NA	NA	68.71	NA	NA	NA
CANVAS Program, 2018 (24)	1461	Canagliflozin	100, 300	3.6	63.78	44.36	12.04	8.40	33.15	NA	NA	72.97	NA	NA	NA
DECLARE-TIMI 58, 2019 (39)	1987	Dapagliflozin	10	4.2	64.16	33.68	10.08	8.27	32.83	48.92	NA	83.57	56.07	7.75	NA
DAPA-HF, 2020 (32)	2139	Dapagliflozin	10	1.5	66.50	22.30	7.76	7.40	29.35	31.20	1482.98	63.35	63.64	35.26	1.00
VERTIS-CV, 2020 (40)	1958	Ertugliflozin	5, 15	3.5	64.37	31.87	12.04	8.30	32.57	NA	NA	NA	65.85	7.08	0.07
SCORED, 2021 (41)	3283	Sotagliflozin	200, 400	1.3	NA	NA	NA	NA	NA	NA	NA	NA	NA	NA	NA
SOLOIST-WHF, 2021 (14, 35)	1222	Sotagliflozin	200	0.8	69.63	33.76	10.59	7.24	30.68	36.00	2006.54	50.37	45.17	45.83	4.42
EMPEROR-reduced, 2021 (37)	1856	Empagliflozin	10	1.3	66.70	23.10	NA	7.40	28.70	27.40	1915.52	61.20	71.40	27.90	0.70
CANONICAL, 2021 (38)	82	Canagliflozin	100	0.5	75.70	32.90	7.08	7.01	25.00	61.50	141.00	57.00	91.50	8.50	NA
EMPEROR-preserved, 2022 (20)	2938	Empagliflozin	10	2.2	70.90	42.80	NA	7.26	31.05	53.90	907.00	59.70	79.40	20.40	0.20
DELIVER, 2022 (25)	2806	Dapagliflozin	10	2.3	71.65	43.90	NA	NA	29.85	54.15	796.12	61.00	75.25	24.45	0.30

DM, diabetes mellitus; HbA1C, glycated hemoglobin; BMI, body mass index; LVEF, left ventricular ejection fraction; NT-proBNP, N-terminal prohormone of B-type natriuretic peptide; eGFR, estimated glomerular filtration rate; NYHA, New York Heart Association. NA, not applicable.

### 3.1 Composite of cardiovascular death or heart failure hospitalization

**Figure 2:**
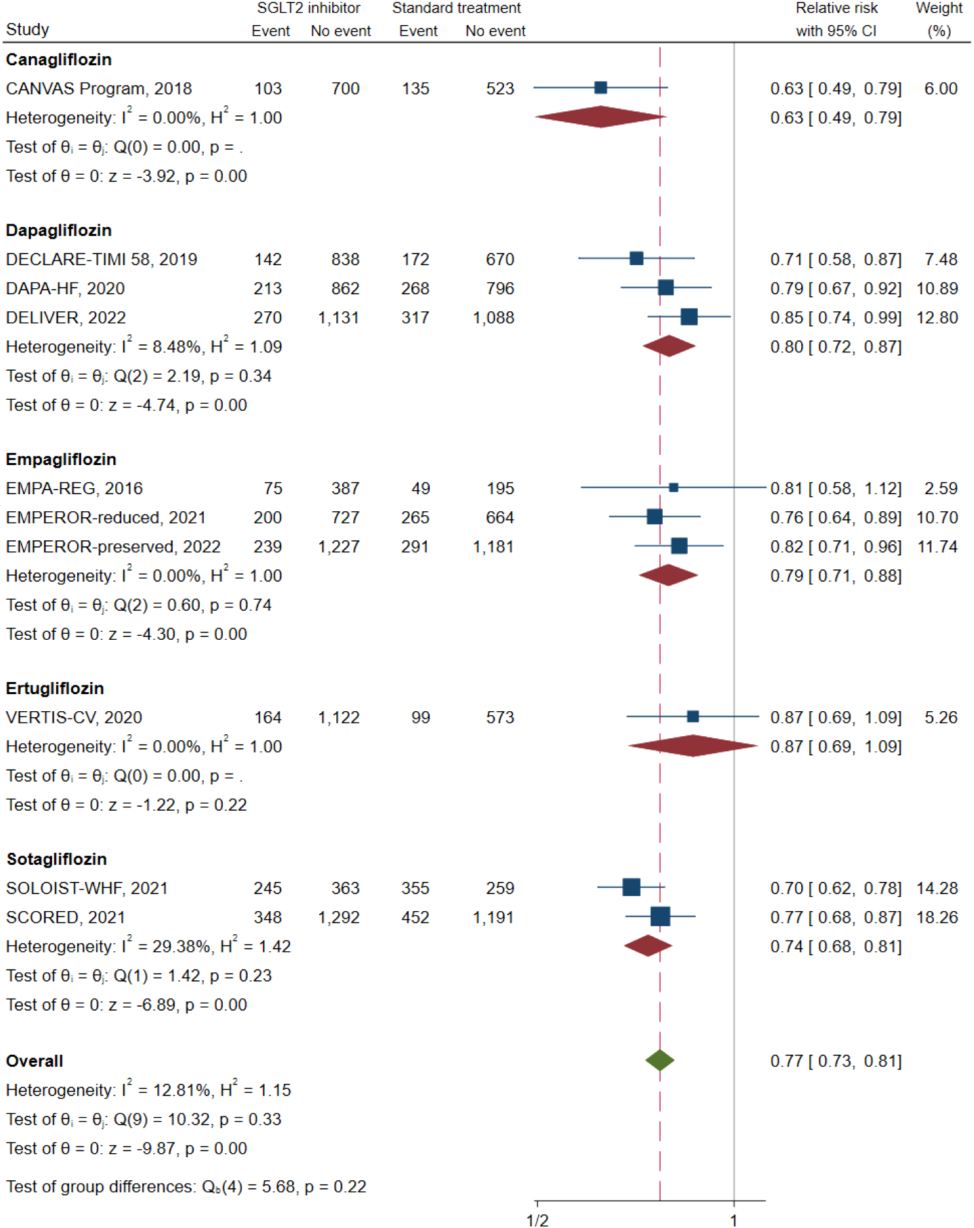
Composite cardiovascular death or heart failure hospitalization in type 2 diabetes with heart failure patients receiving SGLT2 inhibitors versus standard of care. SGLT2, sodium–glucose cotransporter-2.

Ten of the 11 studies (n = 20,191) reported a composite CV death/HFH outcome. A direct MA approach was applied to pool the treatment effects for dapagliflozin (25, 32, 39) (n = 6,932), empagliflozin (20, 31, 37) (n = 5,500), and sotagliflozin (14, 41) (n = 4,505) relative to SoC; canagliflozin (24) (n = 1,461) and ertugliflozin (40) (n = 1,958) were not pooled, as these were single studies. Dapagliflozin, empagliflozin, and sotagliflozin were associated with significant reductions in composite CV death/HFH with pooled RRs (95% CI;I^2^) of 0.80 (0.72–0.87; I^2^ = 8.48%), 0.79 (0.71–0.88; I^2^ = 0.00%), and 0.74 (0.68–0.81; I^2^ = 29.38%), respectively (see **Figure 2**). Overall pooled SGLT2i effects were also significantly associated with reduced composite CV death/HFH with an RR (95% CI) of 0.77 (0.73–0.81; I^2^ = 12.81%).

**Figure 3:**
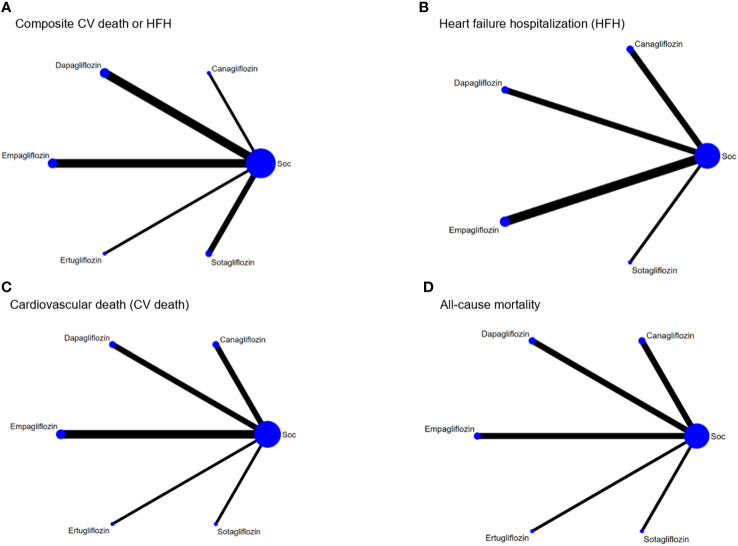
The network plot of the included studies. **(A)** Composite cardiovascular (CV) death or heart failure hospitalization (HFH). **(B)** Heart failure hospitalization (HFH). **(C)** Cardiovascular death (CV death). **(D)** All-cause mortality. The size of the nodes indicates the total sample size of the associated intervention (blue circles). The thickness of each line represents a direct comparison between two therapies and corresponds to the number of trials that examined each comparison.

The NMA (see **Figure 3A**) indicated that with the exception of ertugliflozin, these “add-on” medications were associated with a significantly lowered RR of a composite CV death/HFH outcome between 13% and 37% relative to SoC (see **Table 2**). Of the SGLT2is, both canagliflozin and sotagliflozin had significantly lower composite CV death/HFH compared to dapagliflozin with pooled RRs (95% CI) of 0.75 (0.59, 0.97) and 0.88 (0.78, 1.00), respectively (see **Table 2**). The SUCRA ranking identified “add-on” canagliflozin to SoC as the best for reducing composite CV death/HFH (SUCRA 95.9%), followed by sotagliflozin (SUCRA 77.4%) and empagliflozin (SUCRA 53.2%) (see **Supplementary T** **able S3**, **Supplementary Figure S1A**).

**Table 2:** Relative treatment effect comparison (95% CI) for composite cardiovascular death or heart failure hospitalization (HFH) (upper triangle) and HFH (lower triangle).

Standard of care	0.73* (0.67, 0.80)	0.87 (0.69, 1.09)	0.79* (0.71, 0.88)	0.83* (0.75, 0.91)	0.63* (0.49, 0.79)
0.68* (0.57, 0.81)	Sotagliflozin	1.18 (0.92, 1.51)	1.08 (0.95, 1.24)	1.13 (1.00, 1.28)	0.85 (0.67, 1.10)
		Ertugliflozin	0.92 (0.71, 1.18)	0.96 (0.75, 1.23)	0.72 (0.52, 1.00)
0.71* (0.64, 0.79)	1.05 (0.85, 1.28)		Empagliflozin	1.05 (0.91, 1.20)	0.79 (0.61, 1.02)
0.79* (0.67, 0.93)	1.16 (0.91, 1.48)		1.11 (0.91, 1.35)	Dapagliflozin	0.75* (0.59, 0.97)
0.51*(0.35, 0.74)	0.75 (0.50, 1.13)		0.72 (0.49, 1.06)	0.64* (0.43, 0.97)	Canagliflozin

Comparison should be read from right to left. In the upper rectangle, relative risk <1 favors the drug in the column. In the lower rectangle, relative risk <1 favors the drug in the row.

*Statistical significance.

### 3.2 Heart failure hospitalization

Of the eight studies (n = 12,391) that reported HFH outcomes, two (32, 39) (n = 4,126), one (14) (n = 1,222), two (24, 38) (n = 1,543), and three (20, 31, 37) (n = 5,500) studies investigated dapagliflozin, sotagliflozin, canagliflozin, and empagliflozin, respectively (see **Supplementary Figure S2A**). Canagliflozin, dapagliflozin, and empagliflozin were associated with a significant reduction in HFH relative to SoC with pooled RRs (95% CI; I^2^) of 0.51 (0.35–0.74; I^2^ = 0.00%), 0.79 (0.67–0.93; I^2^ = 0.00%), and 0.71 (0.64–0.79; I^2^ = 11.98%), respectively. Pooling of all SGLT2is provided an RR (95% CI;I^2^) of 0.71 (0.71(0.66-0.76) I^2^ = 6.64%).

The NMA (see **Figure 3B**) indicated that “add-on” canagliflozin, sotagliflozin, empagliflozin, and dapagliflozin treatment led to significant reductions in HFH with RRs (95% CI) of 0.51 (0.35, 0.74), 0.68 (0.57, 0.81), 0.71 (0.64, 0.79), and 0.79 (0.67, 0.93), respectively (**Table 2**). Furthermore, canagliflozin was associated with significant reductions in HFH compared to dapagliflozin [RR (95% CI) 0.64 (0.43, 0.97)]. The SUCRA indicated that the top three ranked SGLT2is were canagliflozin (95.5%), sotagliflozin (66.0%), and empagliflozin (57.2%) (see **Supplementary T** **able S3**, **Supplementary Figure S2B**).

### 3.3 Cardiovascular death

Of the nine studies (n = 14,349) that reported CV death as an outcome, an MA approach was applied across two (32, 39) (n = 4,126), two (24, 38) (n = 1,543), and three (20, 31, 37) (n = 5,500) studies investigating dapagliflozin, canagliflozin, and empagliflozin relative to SoC, respectively (see **Supplementary Figure S3A**). Add-on canagliflozin, dapagliflozin, and empagliflozin were not associated with significantly reduced CV death, with pooled RRs (95% CI; I^2^) of 0.78 (0.57–1.06; I^2^ = 0.00%), 0.89 (0.74–1.06; I^2^ = 40.37%), and 0.92 (0.79–1.09; I^2^ = 0.00%), respectively. However, overall pooling for all SGLT2is was associated with a significant reduction in CV death with an RR (95% CI) of 0.90 (0.81–0.99) without evidence of heterogeneity (I^2^ = 0.00%).

The NMA showed that “add-on” SGLT2is tended to reduce CV death compared to SoC alone, although individually none were significant (see **Table 3**, **Figure 3C**). The top three ranked interventions were canagliflozin (81.5%), dapagliflozin (60.0%), and sotagliflozin (52.9%) (see **Supplementary Table S3**, **Supplementary Figure S3B**).

**Table 3:** Relative treatment effect comparisons (95% CI) for cardiovascular death (upper triangle) and all-cause mortality (lower triangle).

Standard of care	0.89 (0.62, 1.27)	0.95 (0.71, 1.27)	0.92 (0.78, 1.09)	0.88 (0.74, 1.06)	0.78 (0.57, 1.06)
0.86 (0.63, 1.18)	Sotagliflozin	1.07 (0.67, 1.69)	1.04 (0.70, 1.54)	0.99 (0.66, 1.49)	0.87 (0.55, 1.40)
0.97 (0.75, 1.25)	1.12 (0.75, 1.67)	Ertugliflozin	0.98 (0.70, 1.36)	0.93 (0.66, 1.31)	0.82 (0.54, 1.25)
1.01 (0.86, 1.17)	1.17 (0.82, 1.65)	1.04 (0.77, 1.40)	Empagliflozin	0.96 (0.75, 1.22)	0.84 (0.59, 1.19)
0.84* (0.72, 0.98)	0.97 (0.69, 1.38)	0.87 (0.64, 1.17)	0.83 (0.67, 1.04)	Dapagliflozin	0.88 (0.62, 1.26)
0.76 (0.58, 1.00)	0.88 (0.58, 1.33)	0.78 (0.54, 1.14)	0.75 (0.55, 1.03)	0.90 (0.66, 1.24)	Canagliflozin

Comparison should be read from right to left. In the upper rectangle, a relative risk of <1 favors the drug in the column. In the lower rectangle, a relative risk of <1 favors the drug in the row.

*Statistical significance.

### 3.4 All-cause mortality

Of the eight studies (n = 12,493) that reported all-cause mortality outcomes, two (32, 39) (n = 4,126), two (24, 38) (n = 1,543), and two (20, 31) (n = 3,644) studies investigated dapagliflozin, canagliflozin, and empagliflozin relative to SoC, respectively. Only “add-on” dapagliflozin was associated with significant reductions in all-cause mortality with an RR of 0.84 (0.72, 0.98; I^2^ = 0.00), while the remaining SGLT2is were not significant. Collectively, the overall pooled effect of SGLT2is was associated with significantly reduced all-cause mortality with an RR (95% CI) of 0.90 (0.82, 0.99) with no evidence of heterogeneity (I^2^ = 0.00%) (**Supplementary Figure S4A**).

The NMA (**Figure 3D**) showed that “add-on” dapagliflozin and canagliflozin significantly reduced all-cause mortality compared to SoC alone with RRs (95% CI) of 0.84 (0.72, 0.98) and 0.76 (0.58, 1.00) (**Table 3**). The top-ranked interventions by SUCRA were canagliflozin (86.1%), dapagliflozin (72.6%), and sotagliflozin (59.4%) (see **Supplementary Table S3**, **Supplementary Figure S4B**).

### 3.5 Safety outcomes

Seven studies (n = 12,303) reported SAEs associated with dapagliflozin (32, 39) (n = 4,123), sotagliflozin (14) (n = 1,216), canagliflozin (24) (n = 1,461), and empagliflozin (20, 31, 37) (n = 5,503). Dapagliflozin and empagliflozin were associated with a lower risk of SAEs than SoC with pooled RRs (95% CI;I^2^) of 0.87 (0.80–0.95; I^2^ = 0.00%) and 0.89 (0.85–0.94; I^2^ = 0.00%), respectively. The overall pooled SGLT2i RR associated with SAEs compared with SoC was 0.87 (95% CI: 0.83, 0.91), I^2^ = 22.31% (see **Supplementary Figure S5A**). The NMA (**Supplementary Figure S5B**) identified canagliflozin, dapagliflozin, and empagliflozin were associated with a significantly reduced risk of SAEs compared to SoC with RRs (95% CI) of 0.81 (0.75–0.87), 0.87 (0.80–0.95), and 0.89 (0.85–0.94), respectively. Canagliflozin was also borderline significant when compared with empagliflozin [RR 0.90 (0.82–0.99)] and sotagliflozin [RR 0.85 (0.73–1.00)] (see **Supplementary Table S4**) **Supplementary Figure S5C** and **Supplementary Table S3** show the probability of serious AEs of each SGLT2i.

Eight studies (n = 12,377) reported outcomes related to any AEs. Of these, a direct MA approach was applied for canagliflozin (24, 38) (n = 1543), dapagliflozin (32, 39) (n = 4,123), and empagliflozin (20, 31, 37) (n = 5,495). None of the three SGLT2is were significantly associated with any AE outcomes with pooled RRs (95% CI; I^2^) of 1.36 (0.93–1.98; I^2^ = 69.22%), 1.05 (0.96–1.15; I^2^ = 0.00%), and 1.04 (0.91–1.19; I^2^ = 27.08%). The overall pooled RRs across all SGLT2is was 1.07 (95% CI: (0.99-1.15)), I^2^ = 24.98% (see **Supplementary Figure S5E**). The NMA (**Supplementary Figure S5F**) indicated that canagliflozin was associated with a significantly increased risk of any adverse event with RR of (95% CI) of 1.50 (1.01–2.23), 1.45 (0.96–2.2), and 1.42 (0.95–2.13) compared with SoC, empagliflozin, and dapagliflozin, respectively (see **Supplementary Table S5**). Canagliflozin was also associated with the highest probability of any AEs (89.5%), followed by sotagliflozin (71.0%) and dapagliflozin (43.4%) (see **Supplementary Table S3**, **Supplementary Figure S5G**).

**Figure 4:**
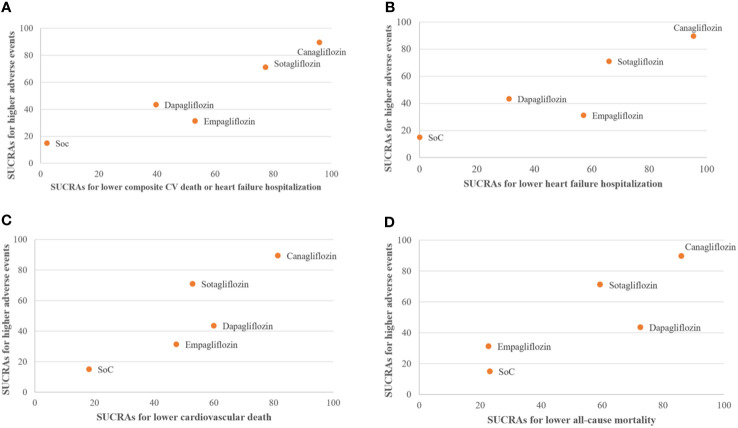
Clustered ranking plot of standard of care (SoC), canagliflozin, dapagliflozin, empagliflozin, and sotagliflozin showing the surface under the cumulative ranking curves (SUCRAs) for the highest probability of any adverse events (AEs) versus the SUCRAs for the highest probability of improving cardiovascular outcomes. **(A)** Composite cardiovascular (CV) death or heart failure hospitalization. **(B)** Heart failure hospitalization. **(C)** CV death. **(D)** All-cause mortality. Intervention lying in the upper right corners are associated with the higher- of probability of treatment efficacy and "higher" probability of AEs.

Clustered ranking suggested that canagliflozin and sotagliflozin offered the best efficacy in reducing HFH and composite CV death/HFH, although both had higher risks associated with any AE. Empagliflozin was associated with a high probability of reducing HFH and the lowest probability of any AEs (**Figure 4**).

### 3.6 Transitivity assessment

Characteristics for each comparison, including age, sex, the strength of the medication, baseline EF, functional class, concurrent medications, and treatment duration, were explored across comparisons (see **Supplementary Table S6**). Age, percentage of females, and EF varied between comparisons. In a network of the composite CV death/HFH outcome, patients in the canagliflozin–SoC comparison were more likely to be younger and female compared to patients in other comparisons. In HFH, CV death, and all-cause mortality networks, patients in the canagliflozin–SoC comparison were more likely to be female and have a higher EF compared to other comparisons. Patients in the ertugliflozin–SoC comparison were more likely to be younger, while those in the sotagliflozin–SoC comparison were more likely to have had a shorter follow-up period for CV death and all-cause mortality networks.

### 3.7 Risk of bias assessment

The RoB was assessed by both reviewers (TK, PK) with 72.73% agreement (kappa 0.48, p = 0.03). Of the 11 RCTs, five were considered low risk, and six had some concerns; the major domain driving this tended to be the randomization process. Given that NMA focused on HF and T2D, studies that did not stratify analyses based on diabetes or HF were considered to have some concerns regarding randomization (**Supplementary Figure S6**).

### 3.8 Publication bias

Comparison-adjusted funnel plots indicated evidence of asymmetry associated with HFH, CV death, and all-cause mortality networks due to small-study effects from a single study (see **Supplementary Figures S1B, S2C, S3C, S4C, S5D, S5H**). However, this was due to a very small effect size.

### 3.9 Sensitivity analysis

Since the HF diagnostic criteria varied between HF-specific RCTs and *post-hoc* analyses of CV death/HFH death outcomes, we performed a sensitivity analysis that included only HF-specific trials at baseline. Consequently, only three SGLT2is, i.e., empagliflozin, dapagliflozin, and sotagliflozin, were retained within the analysis. The results showed that dapagliflozin, empagliflozin, and sotagliflozin significantly reduced composite CV death/HFH with corresponding RRs (95% CI) of 0.82 (0.74–0.92), 0.79 (0.71–0.89), and 0.70 (0.62–0.78); these RRs were comparable with the original analysis that included all HF-specific and T2D-specific trials with corresponding RRs of 0.80 (0.72–0.87), 0.79 (0.71–0.88), and 0.74 (0.68–0.81) (see **Supplementary Figure S7**). A NMA also identified add-on therapies in combination with these three SGLT2is to be significantly associated with reduced risk of composite CV death/HFH compared to SoC. However, no significant differences in head-to-head comparisons were identified (see **Supplementary Table S7**). Similar to our main analysis, SUCRA identified sotagliflozin with the highest probability of reducing composite CV death/HFH (97.6%), followed by empagliflozin (58.4%) and dapagliflozin (44.0%).

In addition, we performed a sensitivity analysis that excluded a single study with a small-study effect (sample size <100) and a treatment duration of less than 6 months (38) from the main analysis. However, our findings remained unchanged (see **Supplementary Fi** **gure S8**).

The Confidence in Network Meta-Analysis (CiNeMA) for each outcome is shown in **Supplementary Table S** **8**. The minimal clinically important differences for each outcome were set according to the Dutch guidelines committee T2D in primary care (42). CiNeMA indicated canagliflozin, sotagliflozin, and empagliflozin had very low confidence ratings for composite CV death/HFH. Within-study bias, reporting bias, and incoherence were the reasons for these downgrades. There was significant concern with incoherence given the lack of a closed loop within the network framework.

## 4 Discussion

A NMA was conducted and revealed that when added to SoC, SGLT2is significantly reduce the composite outcomes of CV death/HFH. Notably, canagliflozin was the most effective, followed by sotagliflozin, while dapagliflozin and empagliflozin exhibited comparable efficacy. The addition of SGLT2is beyond SoC reduced CV death by between 8% and 22%. Only dapagliflozin and canagliflozin were associated with lower all-cause mortality compared to SoC. Importantly, we did not find any statistically significant associations between SGLT2is and adverse side effects or SAEs.

Our findings indicate that SGLT2is reduce composite CV death/HFH outcomes in patients with T2D and previously documented HF by approximately 20%. Although our study encompasses participants from both HF-specific trials and *post-hoc* analyses, our main findings and sensitivity analyses align with those previously reported in an SRMA that focused exclusively on HF-specific trials (43). Notably, the composite outcome of CV death/HFH was primarily influenced by HFH. In our analysis, canagliflozin and sotagliflozin ranked first and second, respectively, in reducing HFH, while they ranked first and third, respectively, in reducing CV death. This ranking is consistent with previous NMA findings (44), which support the notion that non-selective SGLT2is may offer greater advantages in treating HF compared to selective SGLT2is for reducing HFH (44). It is hypothesized that SGLT1 plays a pivotal role in glucose absorption in the intestines, and concurrent inhibition of SGLT1 and SGLT2 may further enhance renal sodium and glucose reabsorption. Furthermore, SGLT1 receptors are expressed in the human myocardium, and their upregulation has been observed in HF patients (45). However, the understanding of the role of SGLT1 cardiac expression and its interactions with SGLT2 in HF patients remains limited.

This study reveals that despite differences in chemical structure, pharmacokinetic and pharmacodynamic properties, as well as variations in SGLT1/SGLT2 receptor selectivity, all SGLT2is investigated in this study generally reduce the risk of HFH, consistent with previous SRMA results (8). We also observed little disparity in the efficacy of individual SGLT2is, with the exception of dapagliflozin, which exhibited a 36% higher rate of HFH compared to canagliflozin. As such, our findings support the beneficial effects of SGLT2is in reducing HFH as a class effect. Notably, the natriuretic and diuretic effects that lead to increased renal glucose excretion may have beneficial implications for endothelial progenitor cells, weight loss, improved myocardial energetics, adaptive cellular reprogramming, and reductions in both blood pressure and left ventricular hypertrophy (46–48).

Previous SRMAs have consistently reported a significant reduction of CV death in patients with T2D who were prescribed SGLT2is (HR 0.85, 95% CI 0.78–0.93, I^2^ = 64.5%, p = 0.02) (8) as well as in patients with HF with or without T2D (HR 0.87, 95% CI 0.79–0.95, p = 0.94) (43). Our study specifically focused on patients with comorbid HF and T2D, and our findings align with the previously reported evidence. Furthermore, our NMA highlights that canagliflozin and dapagliflozin provide the greatest reduction in the risk of CV death, corroborating earlier research (44). Interestingly, we did not observe any significant differences in the ability to reduce CV death between selective and non-selective SGLT2is.

Our findings demonstrate that SGLT2is can reduce all-cause mortality in patients with HF-T2D by approximately 10%. However, only dapagliflozin reached statistical significance, possibly due to the inclusion of two large-scale placebo-controlled RCTs (DECLARE-TIMI and DAPA-HF). The robust reduction in all-cause mortality observed in our study was predominantly driven by the DAPA-HF trial, which revealed a remarkable 17% reduction in all-cause mortality in patients with HF-prescribed dapagliflozin, with or without T2D. In contrast, while the EMPA-REG RCT demonstrated a significant reduction in all-cause mortality, only 9.9% of the patients had a history of cardiac failure at baseline. Moreover, empagliflozin exhibited no survival benefits in the EMPEROR-reduced and EMPEROR-preserved RCTs. Similarly, the impact of canagliflozin, ertugliflozin, and sotagliflozin on mortality outcomes in patients with T2D and HF at baseline was found to be minimal in the CANVAS, VERTIS-CV, and SOLOIST-WHF RCTs, respectively.

The safety profile of SGLT2is is firmly established, encompassing known risks such as mycotic genital infections, urinary tract infections, diabetic ketoacidosis, volume depletion, kidney impairment (16, 19, 23), and the risk of amputation (21). Our findings, as corroborated by our NMA, confirm that SAEs were notably absent across all individual SGLT2is analyzed. However, our analysis did reveal an increased risk of any adverse event associated with canagliflozin.

Although the benefit of SGLT2is in reducing HFH appears to be a class effect, our findings highlight variations among individual SGLT2is in reducing CV and all-cause death, and safety profiles, which may be attributed to several factors. First, each SGLT2i exhibits distinct properties including their selectivity for SGLT1/SGLT2 inhibition, particularly within cardiomyocytes, which could influence CV and renal effects. Second, the differences in the characteristics of the study populations, concomitant medications, the duration of treatments, and follow-up time may introduce elements of heterogeneity, potentially confounding the observed outcomes.

Our NMA has several strengths: first, this is the first NMA to address uncertainties regarding the ranking of CV benefits provided by individual SGLT2is for HF-T2D patients. Second, our NMA includes a broader evidence base, incorporating more RCTs and a larger cohort of HF-T2D patients in comparison to the most recent SRMA (43) (20,438 *vs.* 9,739). Third, we have considered all available SGLT2is (5 SGLT2is *vs.* 3 SGLT2is) and have included additional CV outcome measures, including HFH, CV death, and all-cause mortality in HF-T2D patients. These efforts enable us to comprehensively rank the clinical efficacy and safety profile of individual SGLT2is across all of the CV outcomes of interest.

We also recognize several limitations in our study. First, we employed aggregated study-level data rather than individual patient data, which limited our ability to explore additional baseline factors that might potentially confound outcomes, including concomitant drug used, EF, and the etiology of HF (ischemic or non-ischemic heart disease). Second, our study outcomes may have been influenced by differences in patient populations, study designs, and trial durations. For instance, the SOLOIST-WHF trial focused on T2D patients with more severe HF, enrolling participants either before or within 3 days of HFH, whereas other studies included T2D patients with chronic HF. Variances in the duration of participant follow-up were also observed with CANONICAL and SOLOIST-WHF, which monitored participants for less than 1 year, while other RCTs had longer follow-up periods. Third, our study encompassed both HF-specific and *post-hoc* analysis of CVOTs. We observed disparities in the diagnostic criteria for HF between HF-specific RCTs and *post-hoc* CVOTs. Specifically, all participants enrolled in HF-specific trials exhibited elevated brain natriuretic peptide or NT-proBNP levels, which are established HF diagnostic biomarkers, while diagnostic criteria in CVOTs were less strictly defined. Nevertheless, it is noteworthy that despite these potential confounding factors, the observed heterogeneity in our NMA remained low, and the results from a sensitivity analysis that focused solely on HF-specific trials were consistent with the findings of the overall analysis. Fourth, the efficacy of canagliflozin is primarily derived from the *post-hoc* analysis of CANVAS studies, which did not specifically focus on heart failure at baseline. These results should be interpreted with caution. Fifth, many of the treatment comparisons in our NMA exhibited low confidence levels, as assessed using the six-domain CINeMA tool. These findings underscore the significance of taking into account the uncertainty associated with these comparisons when drawing conclusions from our study.

## 5 Conclusions

SGLT2is significantly reduce the composite CV death/HFH outcome. Among them, canagliflozin may be considered the preferred treatment for patients with diabetes and a history of heart failure, but it may also be associated with an increased risk of any adverse events compared to other SGLT2is. However, a sensitivity analysis focusing on HF-specific trials identified sotagliflozin as the most likely agent to reduce CV death/HFH, followed by empagliflozin and dapagliflozin.

## Data availability statement

The original contributions presented in the study are included in the article/**Supplementary Material**. Further inquiries can be directed to the corresponding author.

## Author contributions

TK contributed to research topic initiation, study design, data collection, data analysis, data interpretation, risk of bias assessment and writing of the manuscript. PK contributed to the risk of bias assessment. PH contributed to the data collection. PL supervised the data collection and risk of bias assessment. VS, SC, UC, GM, and JA provided critical feedback in the writing of the manuscript. AT contributed to research methods, data analysis, data interpretation and provided critical feedback in writing of the manuscript. All authors contributed to the article and approved the submitted version.
