# Supplementary material for: Comparative cardiovascular benefits of individual SGLT2 inhibitors in type 2 diabetes and heart failure: a systematic review and network meta-analysis of randomized controlled trials
Source: Front Endocrinol (Lausanne). 2023 Dec 20;14:1216160. doi: 10.3389/fendo.2023.1216160 (PMC10765518; doi:10.3389/fendo.2023.1216160)
Supplement: Supplementary file 1 [file DataSheet_1.pdf]

frontiers

Supplementary Material

Comparative Cardiovascular Benefits of Individual SGLT2 Inhibitors in Type 2 Diabetes and Heart Failure: A Systematic Review and Network Meta-Analysis of Randomized Controlled trials.

List of supplementary

Supplementary Table S1. Electronic search strategies

Supplementary Table S2. Heart failure medications at baseline

Supplementary Table S3. SUCRAs (%) for highest probability of improving composite cardiovascular (CV) death or heart failure hospitalization (HFH), HFH, CV death and all-cause mortality. SUCRAs for highest probability of serious and any adverse event in patients with type 2 diabetes mellitus and heart failure.

Supplementary 1: Composite CV death or heart failure hospitalization

- **Supplementary Figure S1A.** SUCRA graph of composite CV death or HFH
- **Supplementary Figure S1B.** Comparison-adjusted funnel plots of composite CV death or HFH

Supplementary 2: Heart failure hospitalization

- **Supplementary Figure S2A.** Forest plot of pairwise meta-analysis of HFH
- **Supplementary Figure S2B.** SUCRA graph of heart failure hospitalization
- **Supplementary Figure S2C.** Comparison-adjusted funnel plots of heart failure hospitalization

Supplementary 3: Cardiovascular death

- **Supplementary Figure S3A.** Forest plot of pairwise meta-analysis of CV death
- **Supplementary Figure S3B.** SUCRA graph of cardiovascular death
- **Supplementary Figure S3C.** Comparison-adjusted funnel plots of cardiovascular death

Supplementary 4: All-cause mortality

- **Supplementary Figure S4A.** Forest plot of pairwise meta-analysis of all-cause mortality
- **Supplementary Figure S4B.** SUCRA graph of all-cause mortality
- **Supplementary Figure S4C.** Comparison-adjusted funnel plots of all-cause mortality

Supplementary 5: Safety outcomes

- **Supplementary Figure S5A.** Forest plot of pairwise meta-analysis of serious adverse events
- **Supplementary Figure S5B.** Network plot of serious adverse event

- **Supplementary Table S4.** Relative treatment effects by relative risk on serious adverse event: a network meta-analysis.
- **Supplementary Figure S5C.** SUCRA graph of serious adverse event
- **Supplementary Figure S5D.** Comparison-adjusted funnel plots of serious adverse event
- **Supplementary Figure S5E.** Forest plot of pairwise meta-analysis of any adverse events
- **Supplementary Figure S5F.** Network plot of serious adverse event
- **Supplementary Table S5.** Relative treatment effects by relative risk on any adverse event: a network meta-analysis
- **Supplementary Figure S5G.** SUCRA graph of any adverse event
- **Supplementary Figure S5H**. Comparison-adjusted funnel plots of any adverse event

Supplementary Figure S6: Risk of bias assessment for studies included.

Supplementary Table S6. Transitivity assessment of all networks

Supplementary Figure S7. Forest plot of pairwise meta-analysis which included only HF-specific trials.

Supplementary Table S7. Relative treatment effect on composite cardiovascular death or heart failure hospitalization in sensitivity analysis that included only HF-specific trials.

Supplementary Figure S8. Forest plot of pairwise meta-analysis after exclusion of a study with small-study effect.

Supplementary Table S8: Confidence in Network Meta-Analysis of individual cardiovascular outcome

Supplementary Table S1. Electronic search strategies

1.1 MEDLINE via the PubMed

Domain	No.	Queries	Result
Population (P)	#1	“heart failure” [MeSH Terms]	142,035
	#2	“heart failure"	248,598
	#3	"cardiac failure"	12,797
	#4	“CHF”	16,803
	#5	“HF”	64,924
	#6	“diabetes”	819,556
	#7	“diabetic”	299,291
	#8	“diabetes mellitus”	510,549
	#9	"type 2 diabetes mellitus”	182,176
	#10	“T2DM”	29,756
	#12	“Diabetes Mellitus”[Mesh]	491,155
	#13	#1 OR #2 OR #3 OR #4 OR #5 OR #6 OR #7 OR #8 OR #9 OR #10 OR #11 OR #12	**1,148,416**
Intervention (I)	#14	"Sodium-Glucose Transporter 2 Inhibitors"[Mesh]	5,001
	#15	"SGLT2 inhibitors"	3,163
	#16	"sodium-glucose cotransporter-2 inhibitors"	1,895
	#17	"sodium-glucose transporter 2"[MeSH]	1,627
	#18	"empagliflozin"	2,476
	#19	"canagliflozin"	1,643
	#20	"Tofogliflozin"	148
	#21	"Luseogliflozin”	138
	#22	"Dapagliflozin"	2,341
	#23	"Ertugliflozin"	228
	#24	"Ipragliflozin"	284
	#25	"bexagliflozin"	12
	#26	"henagliflozin"	9
	#27	"Licogliflozin"	15

	#28	"remogliflozin"	44
	#29	"sergliflozin"	18
	#30	"sotagliflozin"	175
	#31	#14 OR #15 OR #16 OR #17 OR #18 OR #19 OR #20 OR #21 OR #22 OR #23 OR #24 OR #25 OR#26 OR #27 OR #28 OR #29 OR #30	**9,015**
Outcome (O)	#32	"Heart failure hospitalization"	1,732
	#33	"death"	951,387
	#34	mortality [MeSH]	420,490
	#35	"all-cause mortality"	47,516
	#36	"cardiovascular mortality"	16,723
	#37	#32 OR #33 OR #34 OR #35 OR #36	**1,284,306**
P & I & O	#38	#13 AND #31 AND #37	1,393
	#39	#38 Filters randomized control trial	**1,257**

1.2 EMBASE (Ovid)

Domain	No.	Queries	Result
Population (P)	#1	'heart failure'/exp OR 'heart failure' OR 'cardiac failure' OR HF OR CHF	741,761
	#2	diabetes OR diabetic OR 'diabetes mellitus' OR 'type 2 diabetes mellitus' OR T2DM OR 'Diabetes Mellitus'/exp	1,515,885
	#3	#1 OR #2	98,592
Intervention (I)	#4	'Sodium glucose cotransporter 2 inhibitor'/exp OR 'SGLT2 inhibitors' OR 'sodium-glucose cotransporter-2 inhibitors' OR 'sodium glucose cotransporter 2'/exp	22,126
	#5	empagliflozin	6,951
	#6	canagliflozin	4,986
	#7	tofogliflozin	496
	#8	luseogliflozin	546
	#9	dapagliflozin	6,846
	#10	ertugliflozin	955

	#11	ipragliflozin	851
	#12	bexagliflozin	23
	#13	henagliflozin	12
	#14	licogliflozin	57
	#15	remogliflozin	237
	#16	‘sergliflozin etabonate’	131
	#17	sotagliflozin	664
	#18	#4 OR #5 OR #6 OR #7 OR #8 OR #9 OR #10 OR #11 OR #12 OR #13 OR #14 OR #15 OR #16 OR #17 OR #18	22,372
Outcome (o)	#19	'heart failure hospitalization'	3,240
	#20	death	1,523,620
	#21	‘mortality’/exp	1,321,552
	#22	‘all-cause mortality’	101,224
	#23	‘Cardiovascular mortality’	68,720
	#24	#19 OR #20 OR #21 OR #22 OR #23	2,531,700
P & I & O	#25	#3 AND #18 AND #24	22,523
S	#26	'randomized controlled trial'/exp OR 'controlled trial, randomized' OR 'randomised controlled study' OR 'randomised controlled trial' OR 'randomized controlled study' OR 'randomized controlled trial' OR 'trial, randomized controlled' OR random*	2,113,375
	#27	#25 AND #26	5,222

1.3 Cochrane central

Domain	No.	Queries	Result
Population (P)	#1	exp Heart Failure/ OR "heart failure" OR "cardiac failure" OR HF OR CHF	37,950

	#2	diabetes OR diabetic OR "diabetes mellitus" OR "type 2 diabetes mellitus" OR T2DM OR exp Diabetes Mellitus/	109,794
	#3	#1 OR #2	142,229
Intervention (I)	#4	exp Sodium-Glucose Transporter 2 Inhibitors/ OR "SGLT2 inhibitors".af. OR "sodium-glucose cotransporter-2 inhibitors".af.	1,313
	#5	empagliflozin.af. OR Tofogliflozin.af. OR canagliflozin.af. OR Luseogliflozin.af. OR Dapagliflozin.af. OR Ertugliflozin.af. OR Ipragliflozin.af. OR bexagliflozin.af. OR henagliflozin.af. OR Licogliflozin.af. OR remogliflozin.af. OR sergliflozin.af. OR sotagliflozin.af.	4,463
	#6	#4 OR #5	4,345
Outcome (o)	#7	Heart failure hospitalization	1,046
	#20	death	83,728
	#21	mortality	119,915
	#22	all-cause mortality	13,751
	#23	cardiovascular mortality	4,804
	#24	#19 OR #20 OR #21 OR #22 OR #23	90,805
P & I & O	#25	#3 AND #18 AND #24	8,550
S	#26	randomized controlled trial	574,665
	#27	#25 AND #26	1,470

Supplementary Table S2. Heart failure medications at baseline (%)

Study	Loop diuretic	Any diuretic	ß-blocker	ACEI or ARB	MRA	ARNI
DAPA-HF	94.8		96.7	83.5	71.5	10.7
SOLOIST-WHF	95.0	10.5	92.1	82.7	64.5	16.8
DECLARE-TIMI 58	40.6	15.5	87.5	86.6	22.1	-
CANVAS Program	25.9	60.1	70.4	85.7		-
VERTIS-CV	29.9	27.6	79.3	84.6	18.3	-
SCORED	35.5	30.0	62.5	87.2	15.0	1.2
EMPEROR-preserved	83.4		87.9	81.8	39.1	2.1
EMPEROR-reduced	89.4		94.7	69.8	70.1	19.2
EMPA-REG	43.0		65.0	81.0	6.0	-
CANONICAL	43.9	-	70.7	84.1	17.1	-
DELIVER	-	-	-	-	-	-

Supplementary Table S3. SUCRAs (%) for highest probability of improving composite cardiovascular (CV) death or heart failure hospitalization (HFH), HFH, CV death and all-cause mortality. SUCRAs for highest probability of serious and any adverse event in patients with type 2 diabetes mellitus and heart failure.

Treatment	Composite CV death or HFH	HFH	CV death	All-cause mortality	Serious adverse events	Any adverse event
Standard of care	2.2	0.1	18.2	23.2	94.7	14.9
Canagliflozin	95.9	95.5	81.5	86.1	3.6	89.5
Dapagliflozin	39.7	31.2	60.0	72.6	34.4	43.4
Empagliflozin	53.2	57.2	47.5	22.8	47.0	31.2
Ertugliflozin	31.5	-	39.8	35.8	-	-
Sotagliflozin	77.4	66.0	52.9	59.4	70.3	71.0

Supplementary 1: Composite CV death or HFH

- **Supplementary Figure S1A.** SUCRA graph of composite CV death or HFH

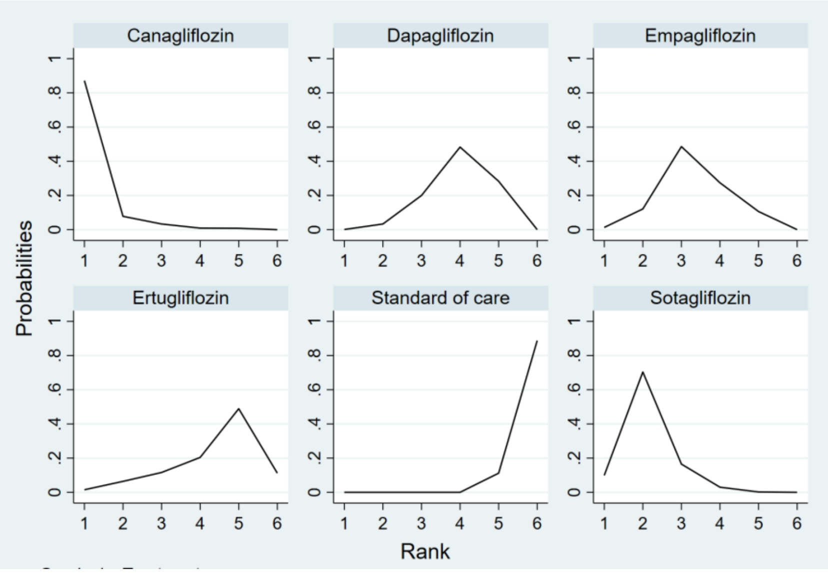

- **Supplementary Figure S1B.** Comparison-adjusted funnel plots of composite CV death or HFH

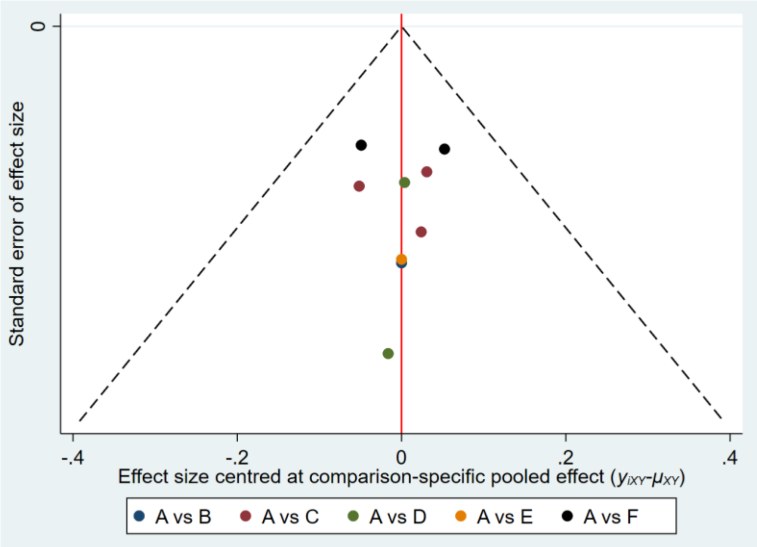

A: standard of care, B. canagliflozin, C. dapagliflozin, D. empagliflozin, E. sotagliflozin

Supplementary 2: Heart failure hospitalization

- **Supplementary Figure S2A.** Forest plot of pairwise meta-analysis of HFH

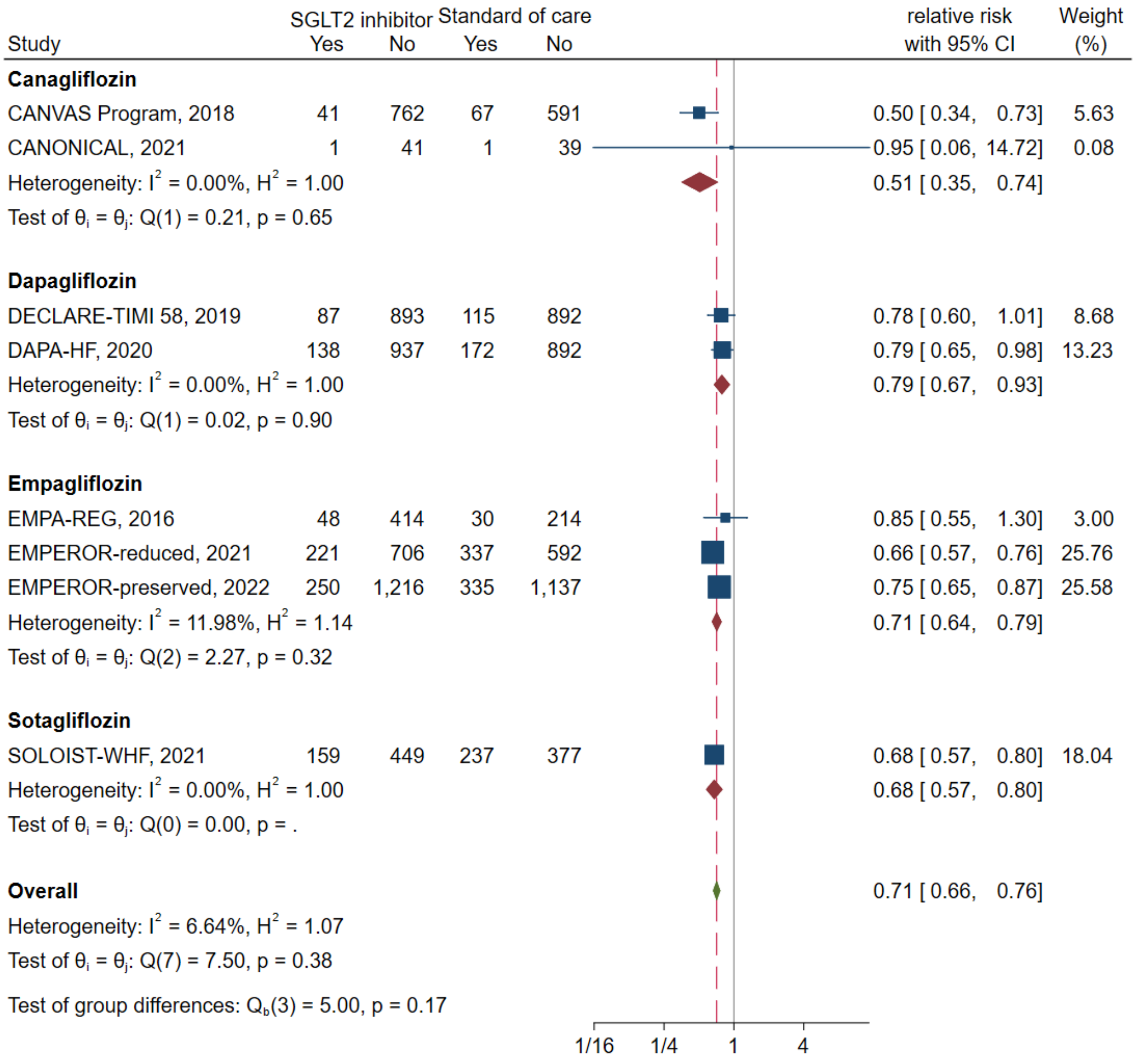

- **Supplementary Figure S2B.** SUCRA graph of heart failure hospitalization

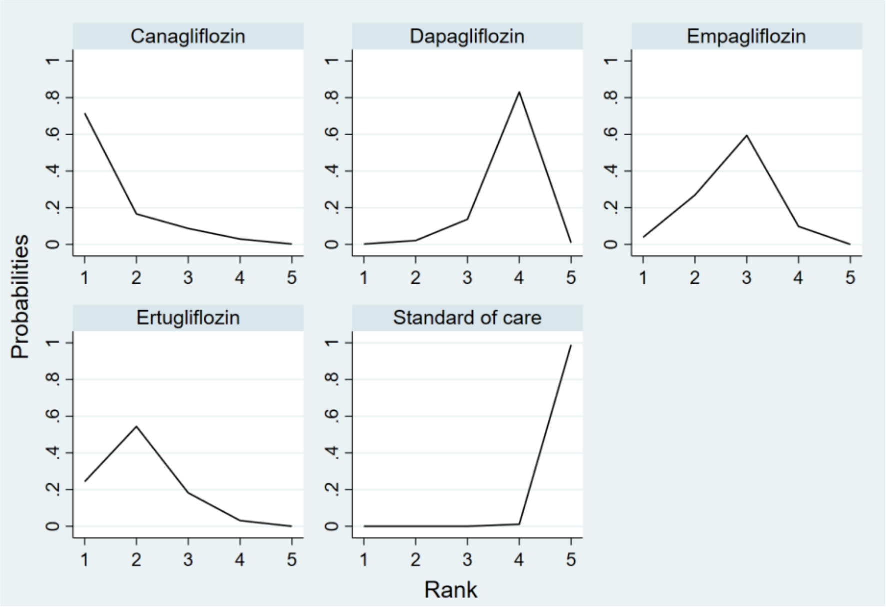

- **Supplementary Figure S2C.** Comparison-adjusted funnel plots of heart failure hospitalization

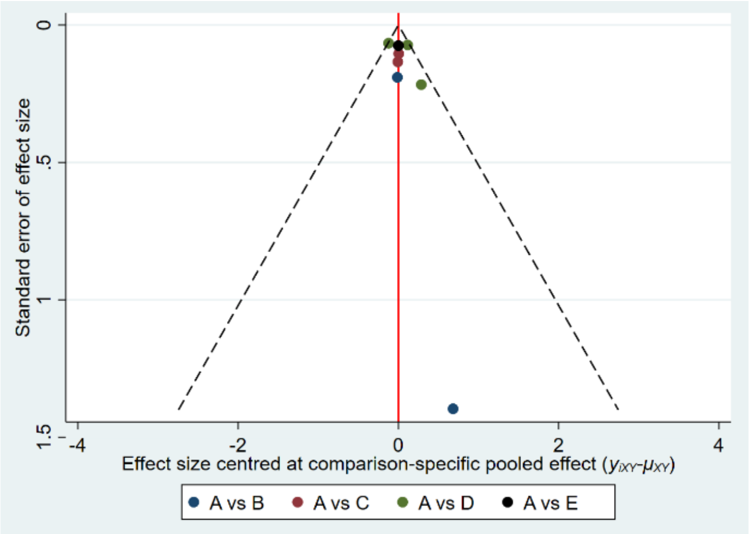

A. standard of care, B. canagliflozin, C. dapagliflozin, D. empagliflozin, E. sotagliflozin

Supplementary 3: Cardiovascular death

- **Supplementary Figure S3A** Forest plot of pairwise meta-analysis of CV death

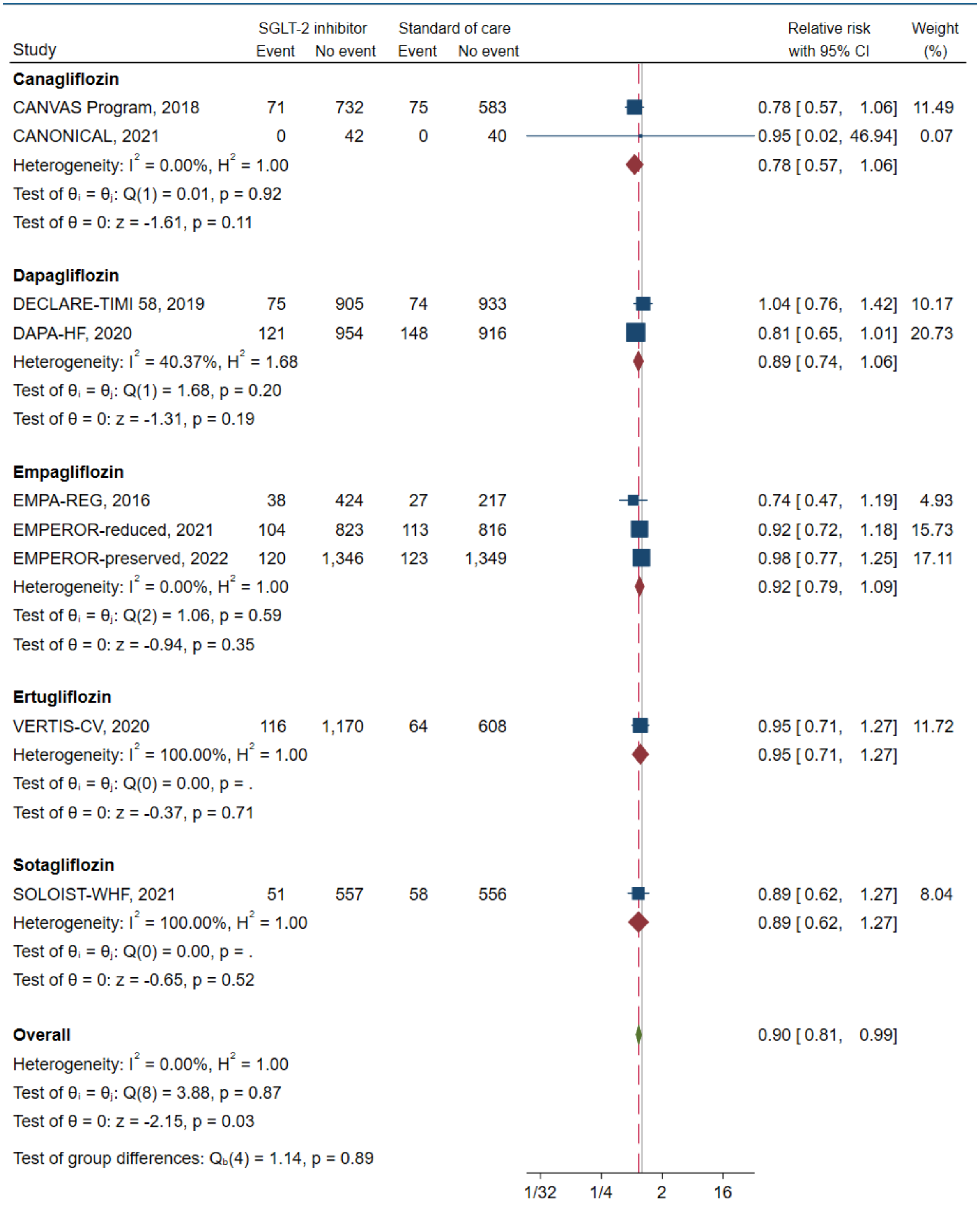

- **Supplementary Figure S3B.** SUCRA graph of cardiovascular death

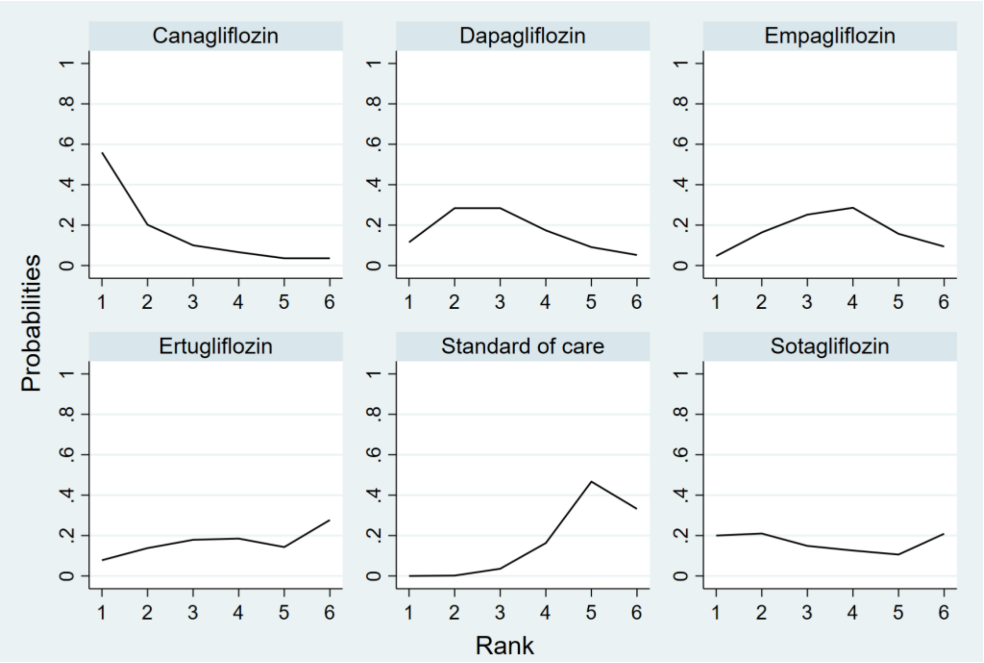

- **Supplementary Figure S3C.** Comparison-adjusted funnel plots of cardiovascular death

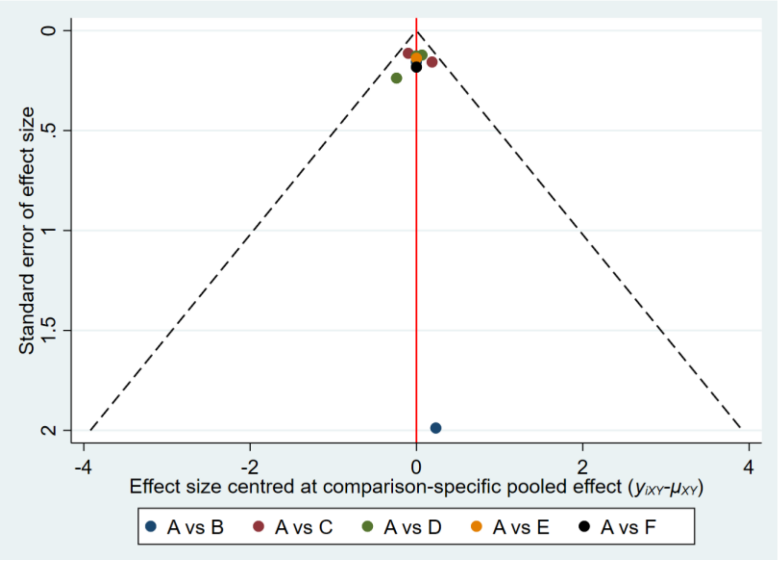

A: standard of care, B. canagliflozin, C. dapagliflozin, D. empagliflozin, E. ertugliflozin, F. sotagliflozin

Supplementary 4: All-cause mortality

- **Supplementary Figure S4A.** Forest plot of pairwise meta-analysis of all-cause mortality

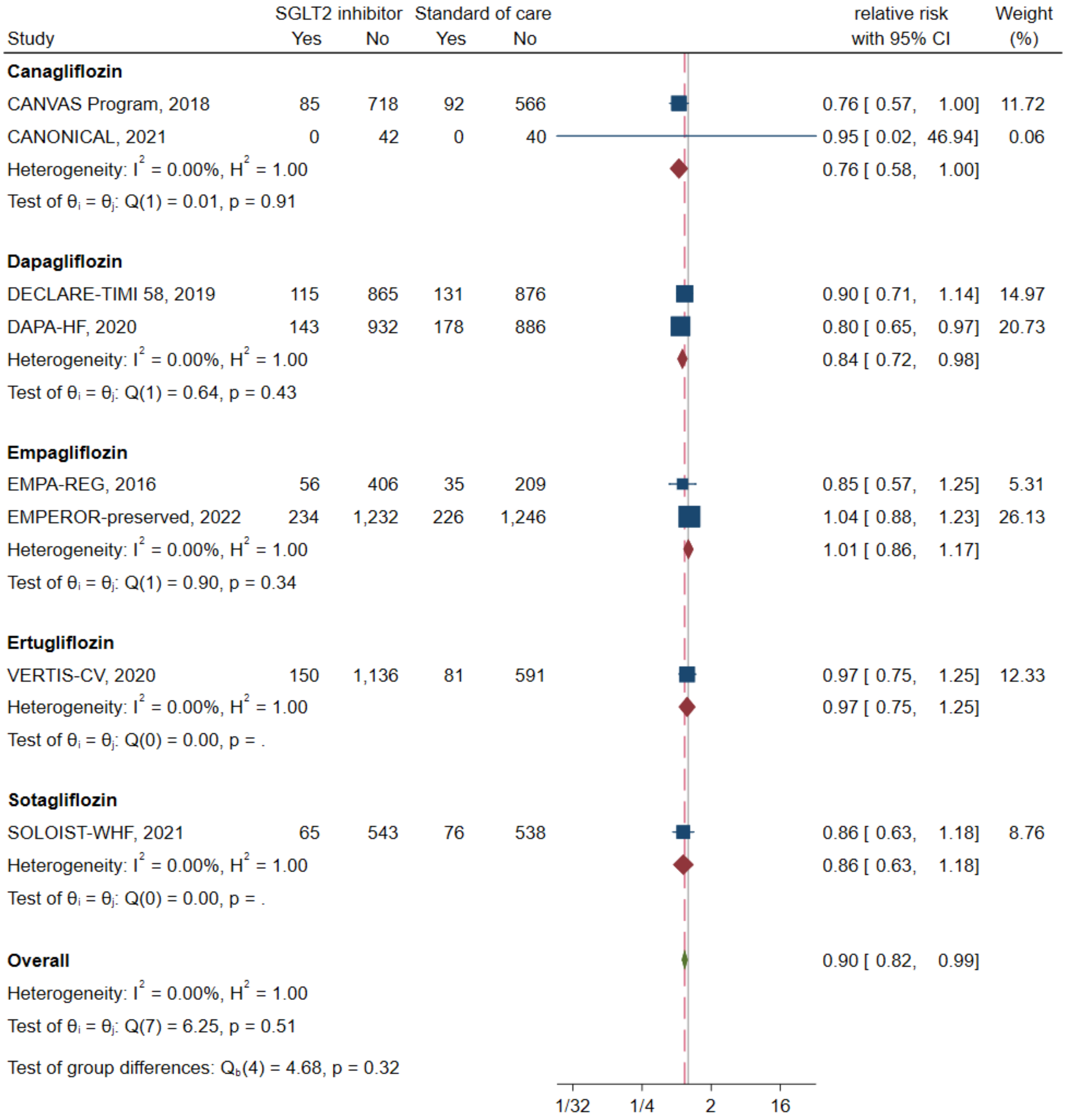

- **Supplementary Figure S4B.** SUCRA graph of all-cause mortality

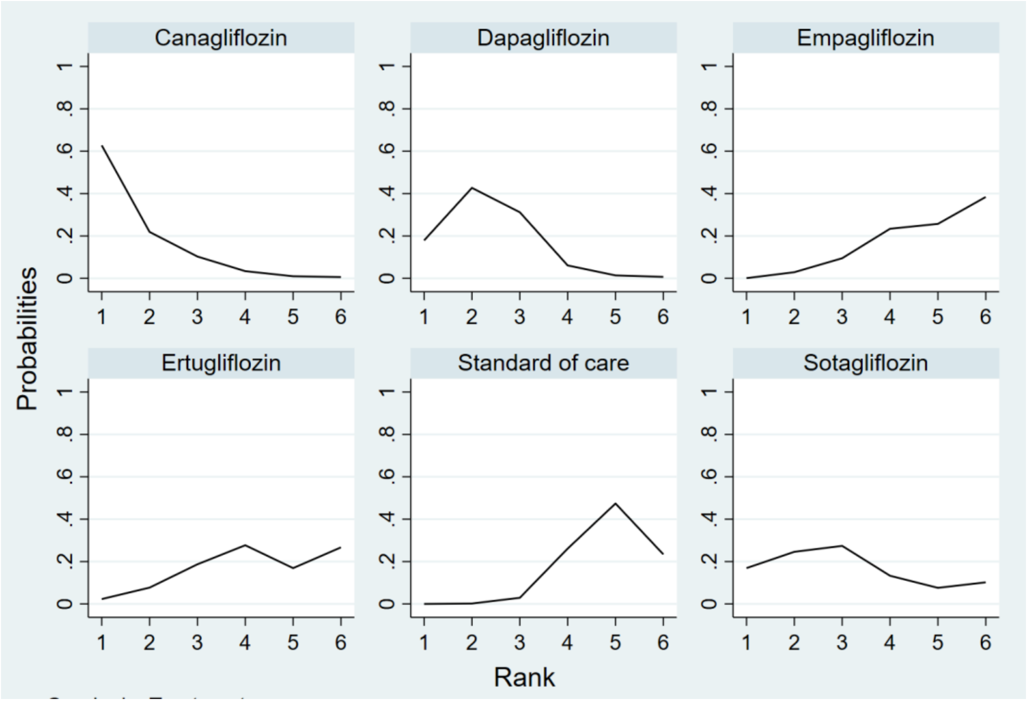

- **Supplementary Figure S4C.** Comparison-adjusted funnel plots of all-cause mortality

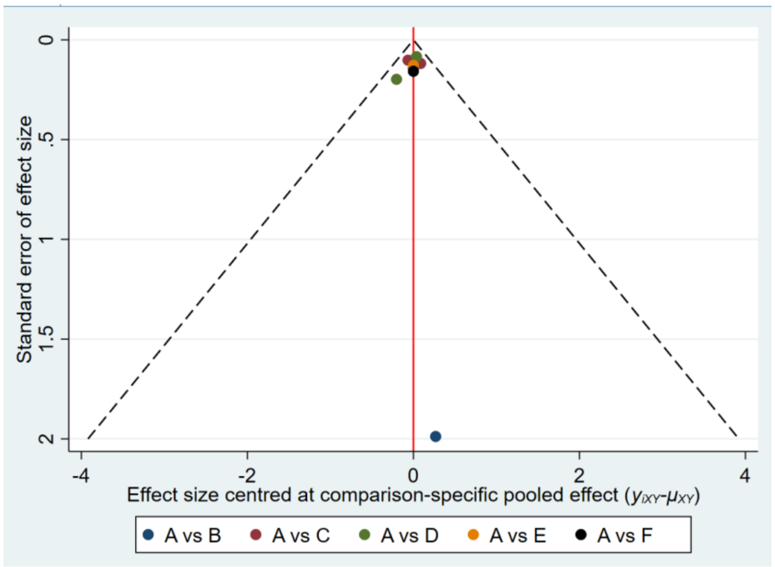

A. standard of care, B. canagliflozin, C. dapagliflozin, D. empagliflozin, E. ertugliflozin, F. sotagliflozin

Supplementary 5: Safety outcomes

- **Supplementary Figure S5A.** Forest plot of pairwise meta-analysis of serious adverse events

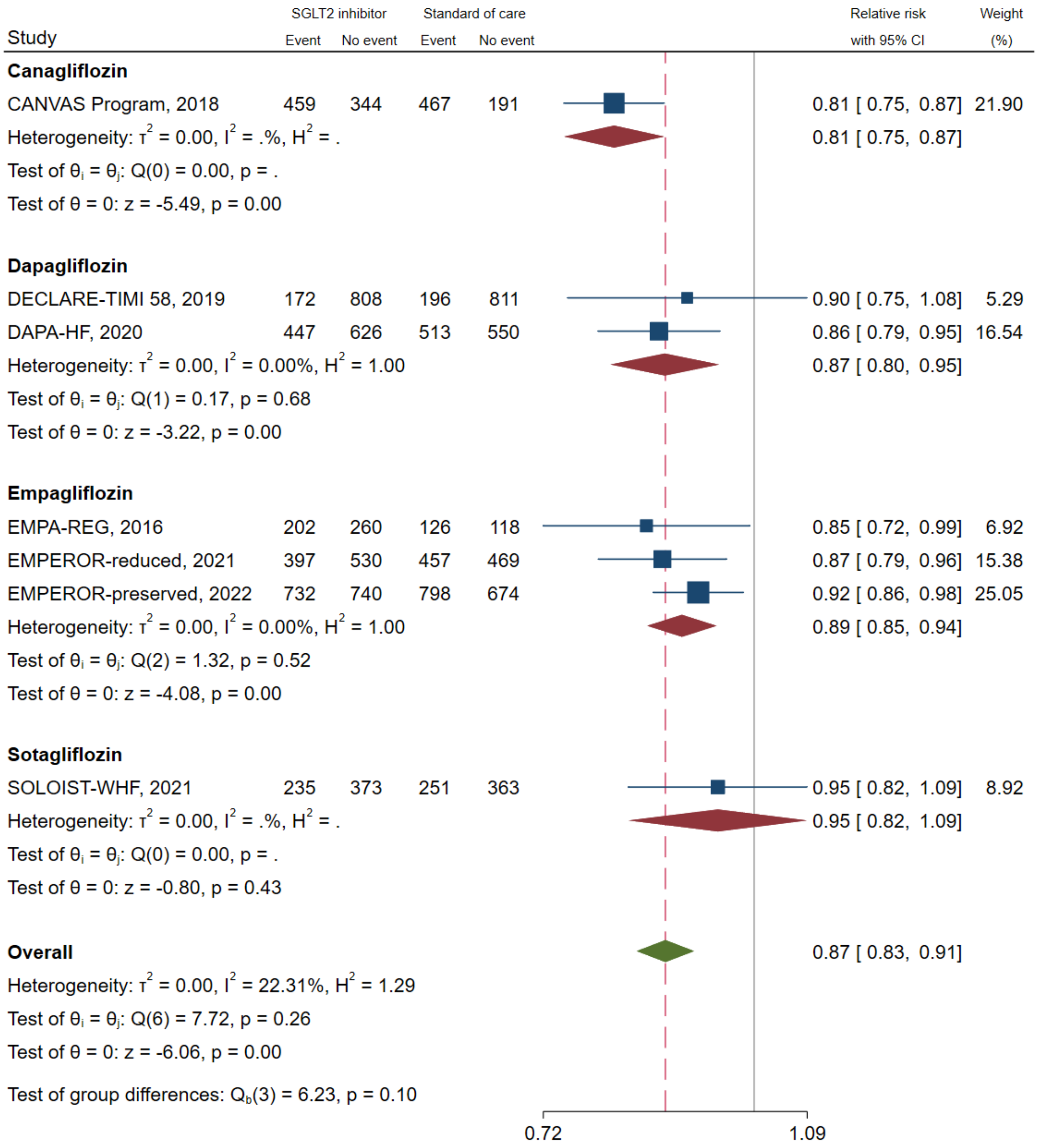

- **Supplementary Figure S5B.** Network plot of serious adverse event

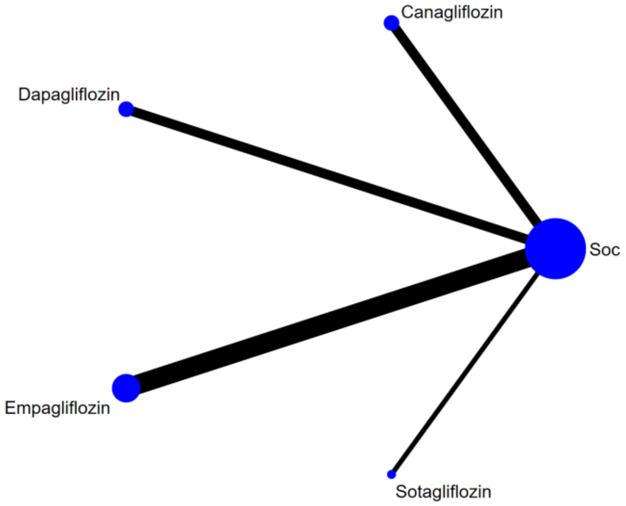

Supplementary Table S4. Relative treatment effects by relative risk on serious adverse event: a network meta-analysis.

Standard of care	0.95 (0.82,1.09)	0.89 (0.85,0.94)	0.87 (0.80,0.95)	0.81 (0.75,0.87)
	Sotagliflozin	0.95 (0.82,1.10)	0.92 (0.78,1.08)	0.85 (0.73,1.00)
		Empagliflozin	0.97 (0.88,1.08)	0.90 (0.82,0.99)
			Dapagliflozin	0.92 (0.82,1.04)
				Canagliflozin

- **Supplementary Figure S5C.** SUCRA graph of serious adverse event

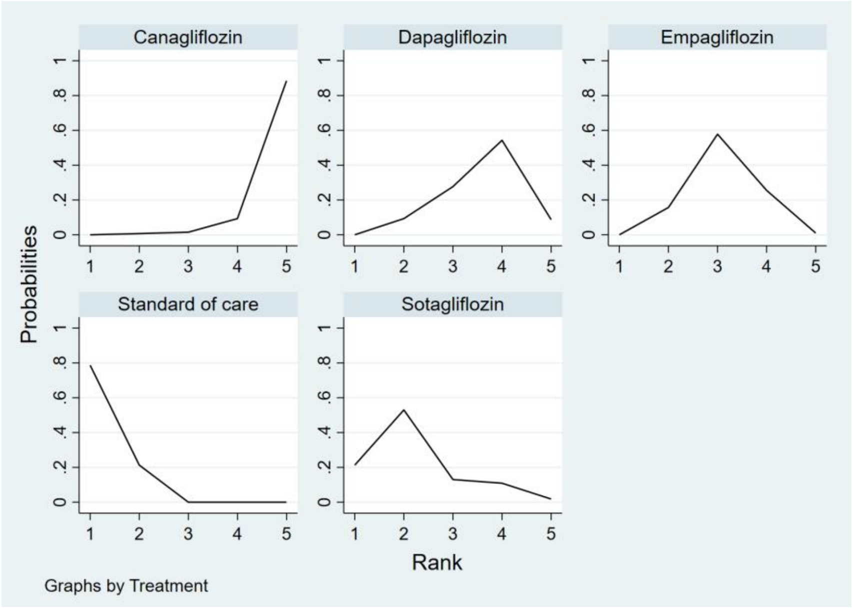

- **Supplementary Figure S5D.** Comparison-adjusted funnel plots of serious adverse event

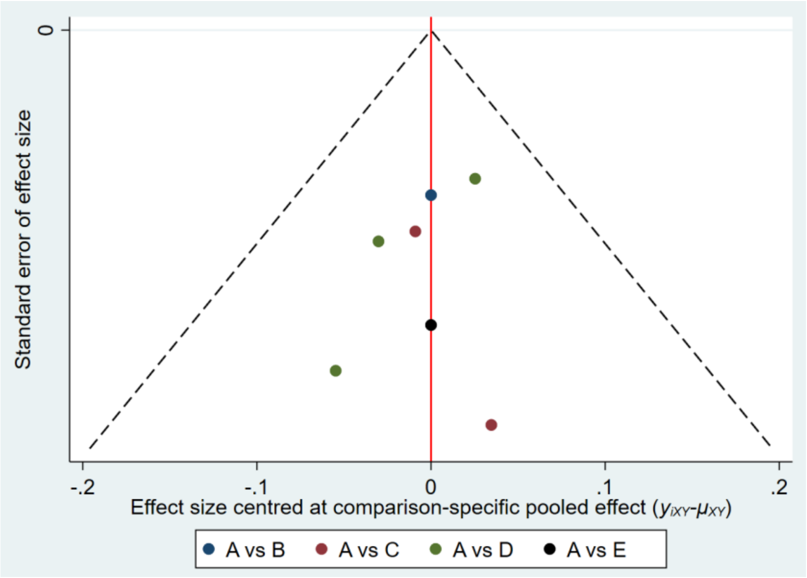

A: standard of care, B. canagliflozin, C. dapagliflozin, D. empagliflozin, E. sotagliflozin

- **Supplementary Figure S5E.** Forest plot of pairwise meta-analysis of any adverse event

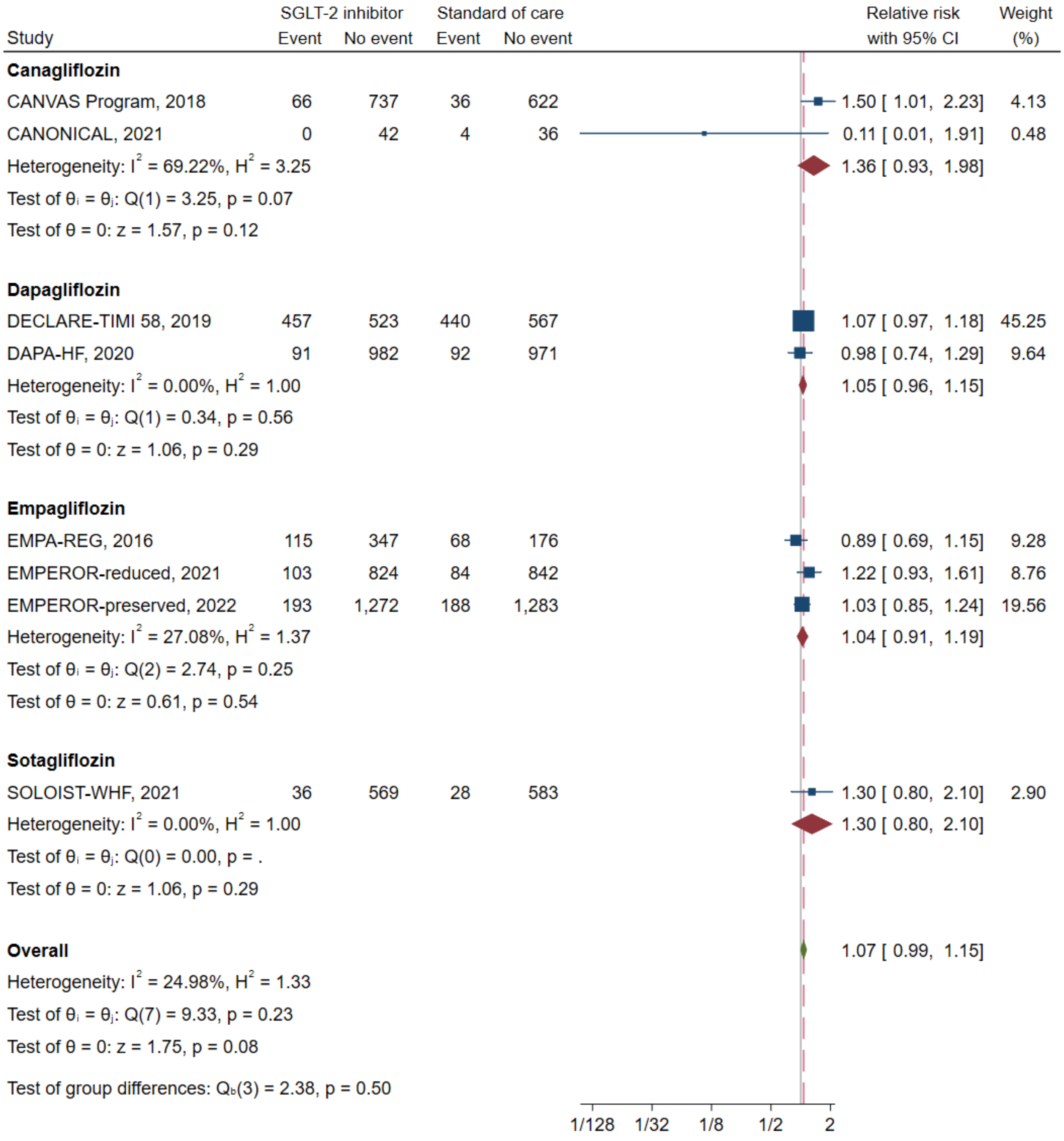

- **Supplementary Figure S5F.** Network plot of any adverse event

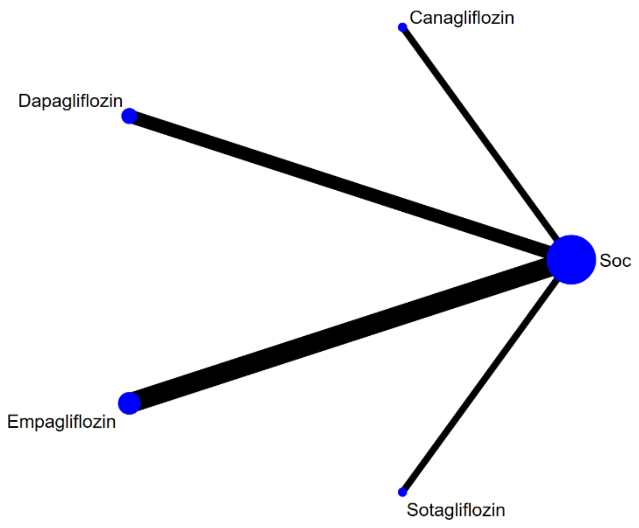

Supplementary Table S5. Relative treatment effects by relative risk on any adverse event: a network meta-analysis.

Standard of care	1.30 (0.80,2.10)	1.03 (0.91,1.18)	1.06 (0.96,1.16)	1.50 (1.01,2.23)
	Sotagliflozin	0.80 (0.48,1.31)	0.81 (0.50,1.33)	1.16 (0.62,2.15)
		Empagliflozin	1.02 (0.87,1.20)	1.45 (0.96,2.20)
			Dapagliflozin	1.42 (0.95,2.13)
				Canagliflozin

- **Supplementary Figure S5G**. SUCRA graph of any adverse event

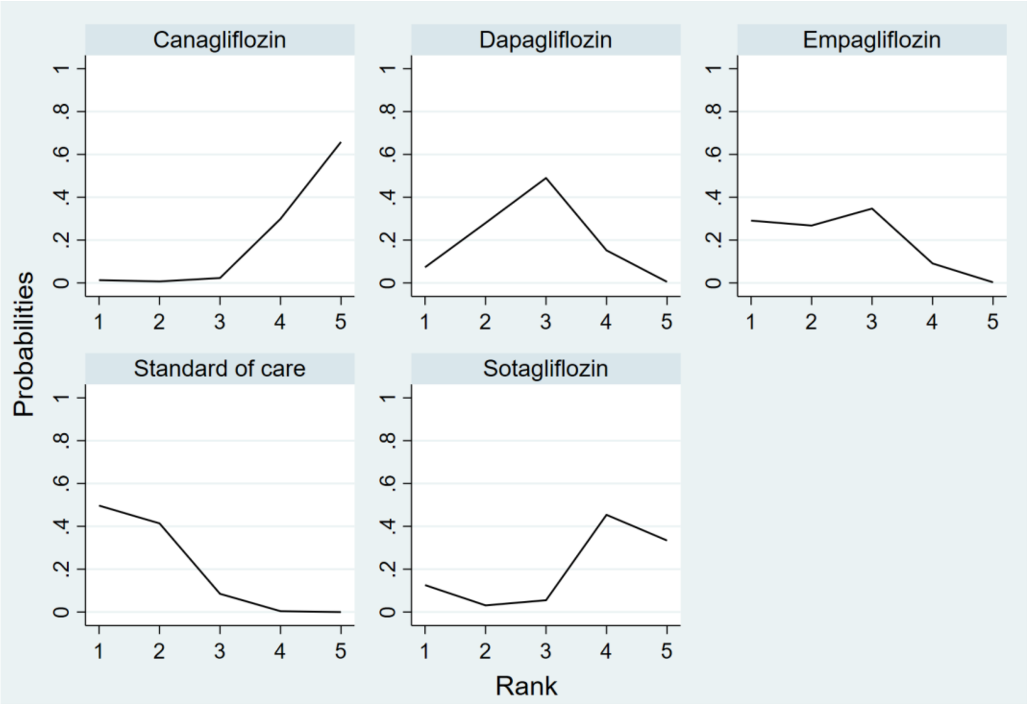

- **Supplementary Figure S5H**. Comparison-adjusted funnel plots of any adverse event

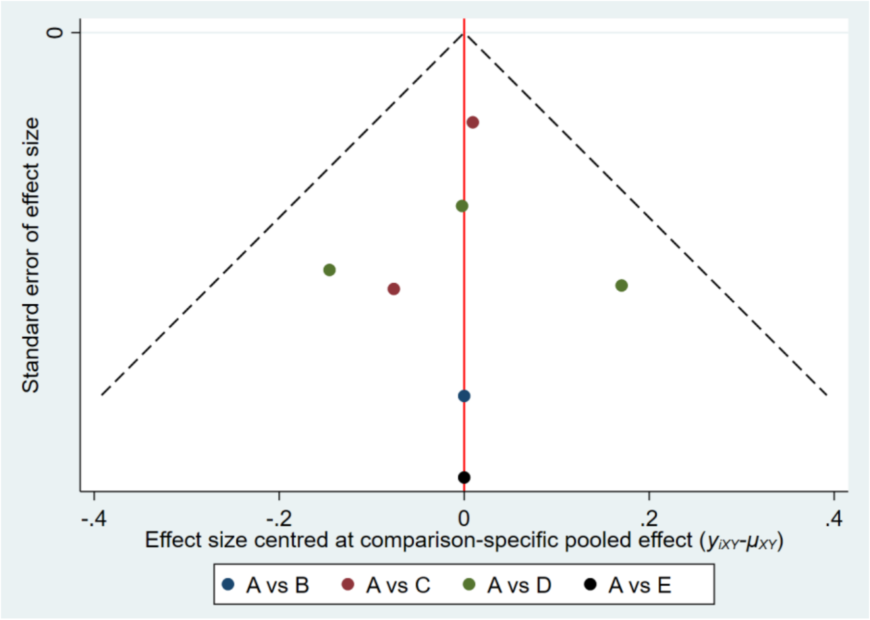

A: standard of care, B. canagliflozin, C. dapagliflozin, D. empagliflozin, E. sotagliflozin

Supplementary Figure S6: Risk of bias assessment for studies included.

Study label	Year	Intervention	Comparator	D1	D2	D3	D4	D5	Overall
EMPA-REG OUTCOME	2016	Empagliflozin	Standard of care	!	+	+	+	+	!
CANVAS program	2018	Canagliflozin	Standard of care	!	+	+	!	+	!
DECLARE-TIMI 58	2019	Dapafliglozin	Standard of care	!	+	+	+	+	!
DAPA-HF	2020	Dapagliflozin	Standard of care	+	+	+	+	+	+
VERTIS-CV	2020	Ertugliflozin	Standard of care	!	+	+	+	+	!
SCORED	2021	Sotagliflozin	Standard of care	!	+	+	+	+	!
SOLOIST-WHF	2021	Sotagliflozin	Standard of care	+	+	+	+	+	+
CANONICAL	2021	Canagliflozin	Standard of care	+	!	+	!	+	!
EMPEROR-reduced	2021	Empagliflozin	Standard of care	+	+	+	+	+	+
EMPEROR-preserved	2022	Empagliflozin	Standard of care	+	+	+	+	+	+
DELIVER	2022	Dapagliflozin	Standard of care	+	+	+	+	+	+

+ Low risk

! Some concerns

\- High risk

D1 Randomisation process

D2 Deviations from the intended interventions

D3 Missing outcome data

D4 Measurement of the outcome

D5 Selection of the reported result

Supplementary Table S6. Transitivity assessment of all networks

	No. of studies	No. of patients	Age	Sex (F%)	Dose of medication	EF (%)	NYHA II (%)	MRA (%)	ARNI (%)	Diuretic (%)	Follow up time (yrs)
**Composite cardiovascular death or heart failure hospitalization**											
Canagliflozin:SoC	1	1,461	63.8	44.4	100, 300	NA	NA	NA	NA	60.1	3.62
Dapagliflozin:SoC	3	6,932	67.4	33.3	10	44.8	65.0	46.8	NA	75.5	2.86
Empagliflozin:SoC	3	5,500	67.4	31.9	10, 25	40.7	75.4	38.4	10.7	71.9	2.2
Ertugliflozin:SoC	1	1,958	64.4	31.9	5, 15	NA	65.9	NA	NA	NA	3.5
Sotagliflozin:Soc	2	4,505	69.6	22.3	200, 400	36	45.2	39.8	16.8	80.3	1.1
**Heart failure hospitalization**											
Canagliflozin:Soc	2	1,543	69.7	38.6	100, 300	61.5	91.5	NA	NA	65.0	2.04
Dapagliflozin:Soc	2	4,126	65.3	28.0	10	40.1	59.9	46.8	NA	75.5	2.9
Empagliflozin:Soc	3	5,500	67.4	31.9	10, 25	40.7	75.4	38.4	10.7	71.9	2.2
Sotagliflozin:Soc	1	1,222	69.6	32.9	200	NA	NA	NA	NA	NA	0.77

**Cardiovascular death**											
Canagliflozin:SoC	2	1,543	69.7	38.6	100, 300	61.5	91.5	NA	NA	65.0	2.04
Dapagliflozin:SoC	2	4,126	65.3	28.0	10	40.1	59.9	46.8	NA	75.5	2.9
Empagliflozin:SoC	3	5,500	67.4	31.9	10, 25	40.7	75.4	38.4	10.7	71.9	2.2
Ertugliflozin:SoC	1	1,958	64.4	31.9	5, 15	NA	65.9	NA	NA	NA	3.5
Sotagliflozin:Soc	1	1,222	69.6	32.9	200	NA	NA	NA	NA	NA	0.77
**All-cause mortality**											
Canagliflozin:SoC	2	1,543	69.7	38.6	100, 300	61.5	91.5	NA	NA	65.0	2.04
Dapagliflozin:SoC	2	4,126	65.3	28.0	10	40.1	59.9	46.8	NA	75.5	2.9
Empagliflozin:SoC	2	3,644	67.7	36.3	10, 25	64.2	79.4	22.6	2.1	63.2	2.6
Ertugliflozin:SoC	1	1,958	64.4	31.9	5, 15	NA	65.9	NA	NA	NA	3.5
Sotagliflozin:Soc	1	1,222	69.6	32.9	200	NA	NA	NA	NA	NA	0.77
**Serious adverse events**											
Canagliflozin:SoC	1	1,461	63.8	44.4	100, 300	NA	NA	NA	NA	60.1	3.62

Dapagliflozin:SoC	2	4,126	65.3	28.0	10	40.1	59.9	46.8	NA	75.5	2.9
Empagliflozin:SoC	3	5,500	67.4	31.9	10, 25	40.7	75.4	38.4	10.7	71.9	2.2
Sotagliflozin:Soc	1	1,222	69.6	32.9	200	NA	NA	NA	NA	NA	0.77
**Any adverse events**											
Canagliflozin:SoC	2	1,543	69.7	38.6	100, 300	61.5	91.5	NA	NA	65.0	2.04
Dapagliflozin:SoC	2	4,126	65.3	28.0	10	40.1	59.9	46.8	NA	75.5	2.9
Empagliflozin:SoC	4	6,206	66.7	31.4	10, 25	40.7	75.4	38.4	10.7	71.9	1.9
Sotagliflozin:Soc	1	1,222	69.6	32.9	200	NA	NA	NA	NA	NA	0.77

Supplementary Figure S7. Forest plot of pairwise meta-analysis which included only HF-specific trails at baseline.

- **Composite cardiovascular death or heart failure hospitalization**

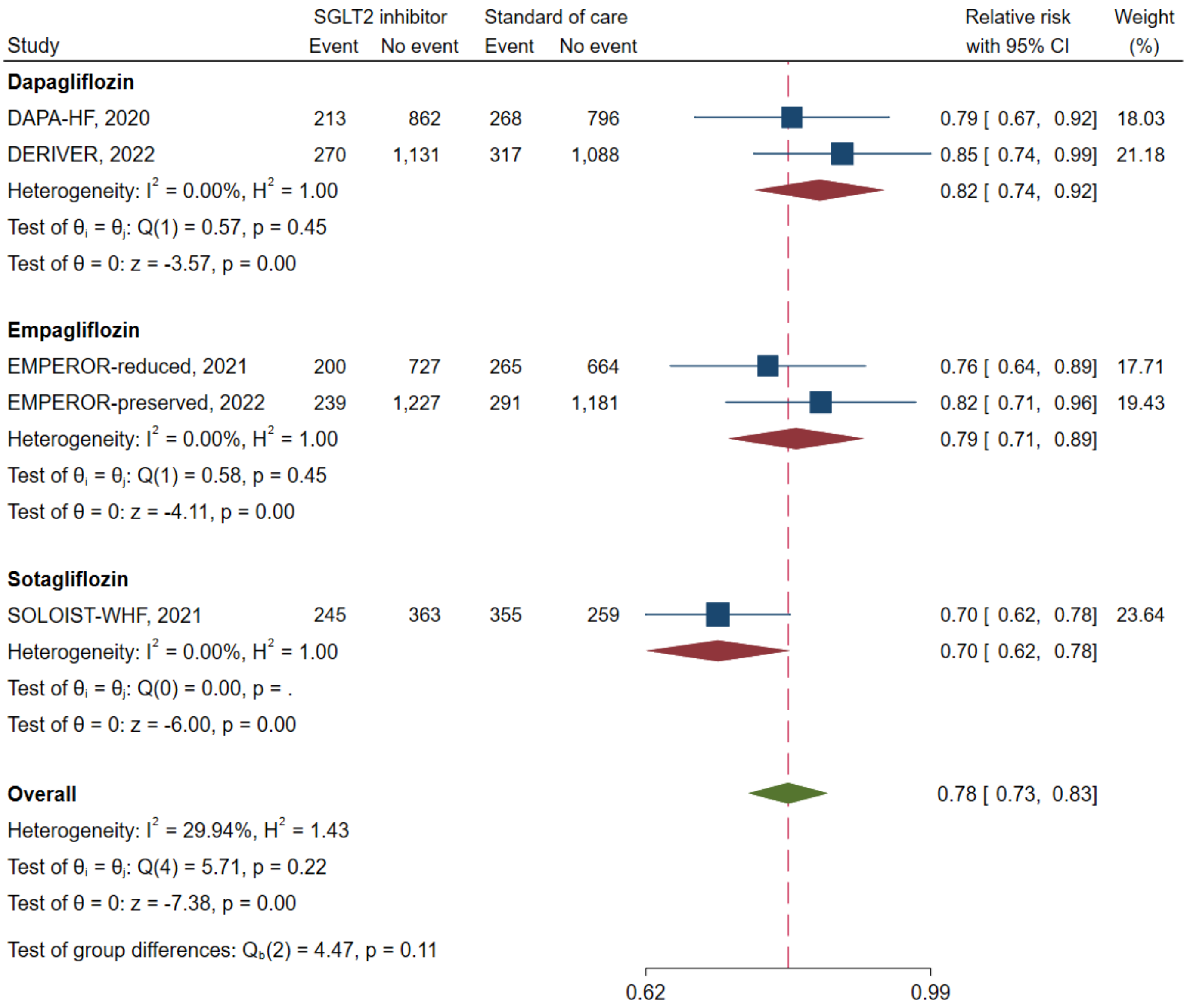

Supplementary Table S7. Relative treatment effect on composite cardiovascular death or heart failure hospitalization in sensitivity analysis that included only HF-specific trials.

Standard of care	0.70 (0.62,0.78)	0.79 (0.71,0.88)	0.82 (0.74,0.92)
	Sotagliflozin	1.13 (0.96,1.33)	1.18 (1.01,1.38)
		Empagliflozin	1.04 (0.89,1.21)
			Dapagliflozin

Supplementary Figure S8. Forest plot of pairwise meta-analysis after exclusion of a study with small-study effect.

- **Heart failure hospitalization**

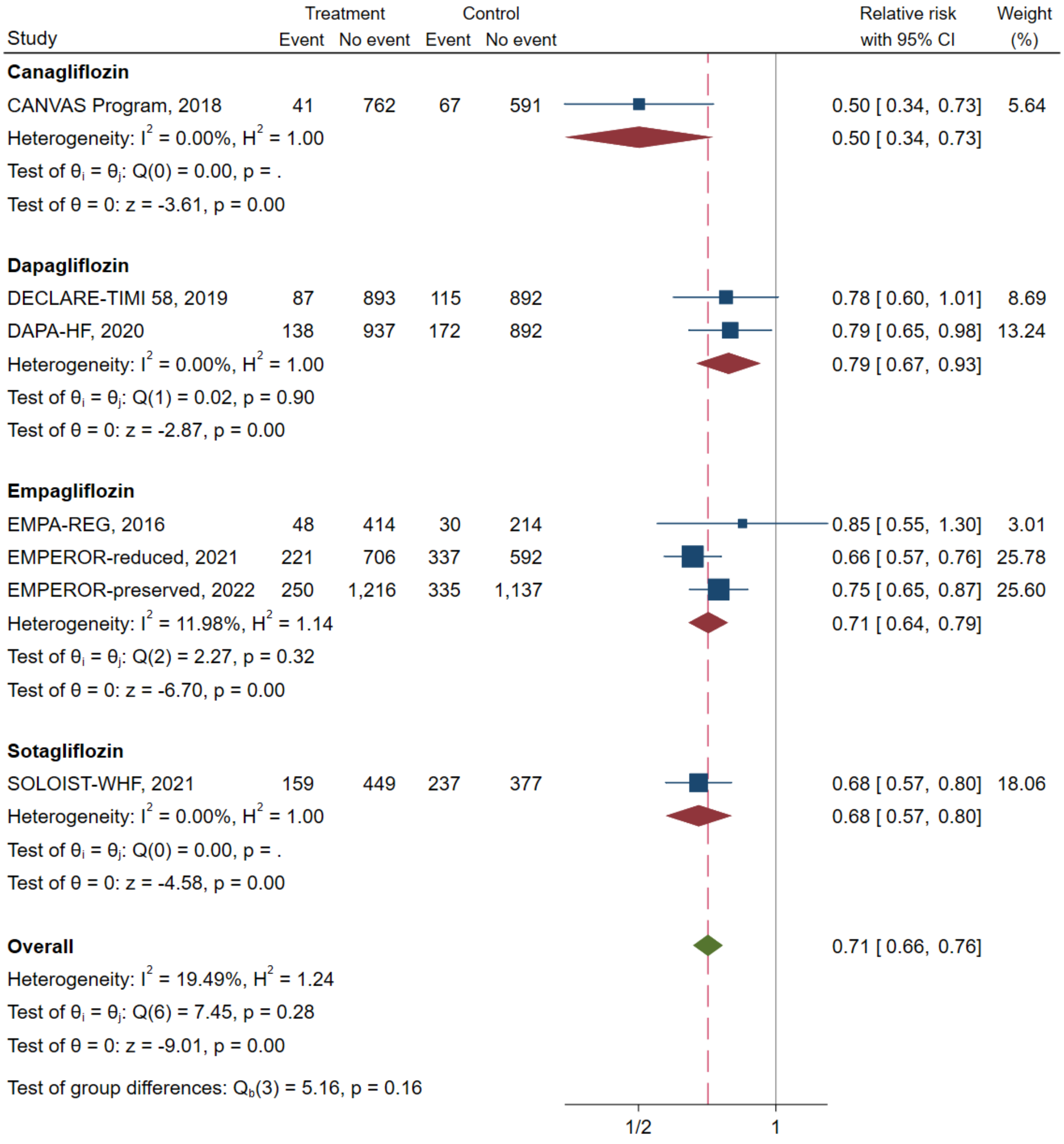

- **Cardiovascular death**

Study	Treatment Event	Treatment No event	Control Event	Control No event	Relative risk with 95% CI	Weight (%)
**Canagliflozin**						
CANVAS Program, 2018	71	732	75	583	0.78 [0.57, 1.06]	11.50
Heterogeneity: I^2 = 0.00%, H^2 = 1.00					0.78 [0.57, 1.06]	
Test of $\theta_i = \theta_j$: Q(0) = 0.00, p = .						
Test of θ = 0: z = -1.62, p = 0.11						
**Dapagliflozin**						
DECLARE-TIMI 58, 2019	75	905	74	933	1.04 [0.76, 1.42]	10.18
DAPA-HF, 2020	121	954	148	916	0.81 [0.65, 1.01]	20.75
Heterogeneity: I^2 = 40.37%, H^2 = 1.68					0.89 [0.74, 1.06]	
Test of $\theta_i = \theta_j$: Q(1) = 1.68, p = 0.20						
Test of θ = 0: z = -1.31, p = 0.19						
**Empagliflozin**						
EMPA-REG, 2016	38	424	27	217	0.74 [0.47, 1.19]	4.93
EMPEROR-reduced, 2021	104	823	113	816	0.92 [0.72, 1.18]	15.74
EMPEROR-preserved, 2022	120	1,346	123	1,349	0.98 [0.77, 1.25]	17.12
Heterogeneity: I^2 = 0.00%, H^2 = 1.00					0.92 [0.79, 1.09]	
Test of $\theta_i = \theta_j$: Q(2) = 1.06, p = 0.59						
Test of θ = 0: z = -0.94, p = 0.35						
**Ertugliflozin**						
VERTIS-CV, 2020	116	1,170	64	608	0.95 [0.71, 1.27]	11.73
Heterogeneity: I^2 = 100.00%, H^2 = 1.00					0.95 [0.71, 1.27]	
Test of $\theta_i = \theta_j$: Q(0) = 0.00, p = .						
Test of θ = 0: z = -0.37, p = 0.71						
**Sotagliflozin**						
SOLOIST-WHF, 2021	51	557	58	556	0.89 [0.62, 1.27]	8.05
Heterogeneity: I^2 = 100.00%, H^2 = 1.00					0.89 [0.62, 1.27]	
Test of $\theta_i = \theta_j$: Q(0) = 0.00, p = .						
Test of θ = 0: z = -0.65, p = 0.52						
**Overall**					0.90 [0.81, 0.99]	
Heterogeneity: I^2 = 0.00%, H^2 = 1.00						
Test of $\theta_i = \theta_j$: Q(7) = 3.87, p = 0.79						
Test of θ = 0: z = -2.15, p = 0.03						
Test of group differences: $Q_b(4)$ = 1.15, p = 0.89						

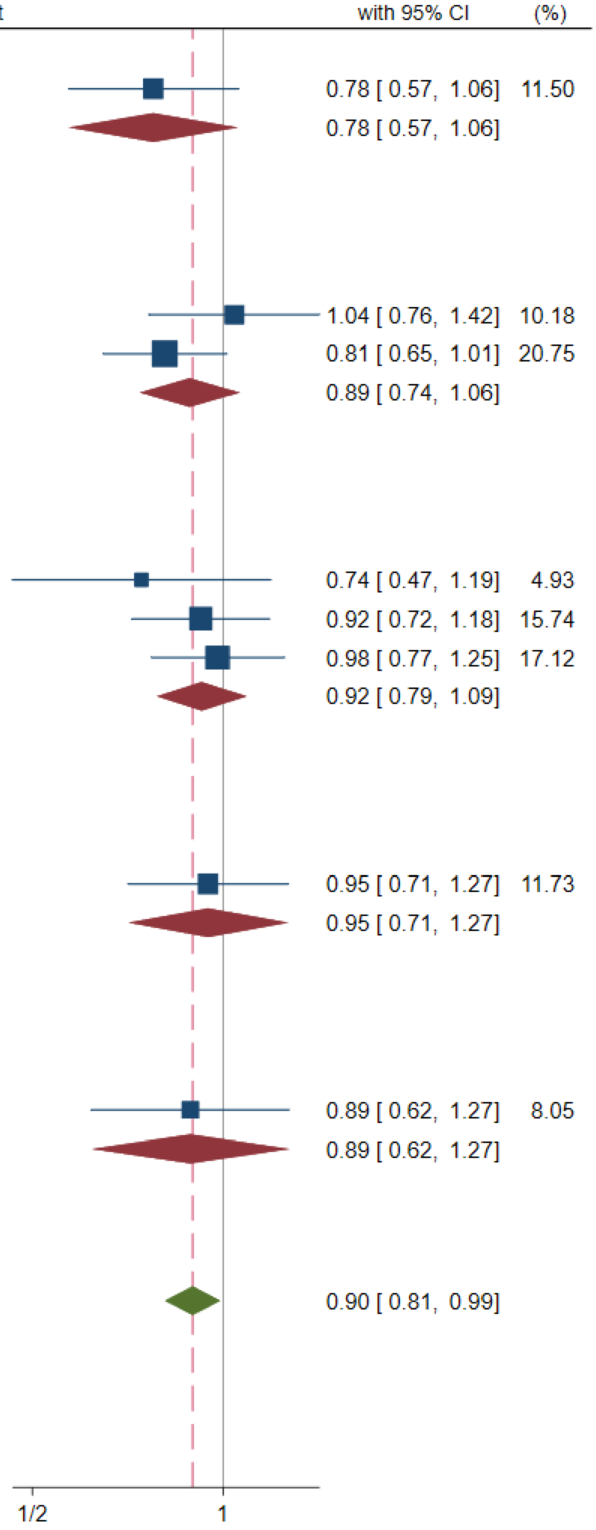

- **All -cause mortality**

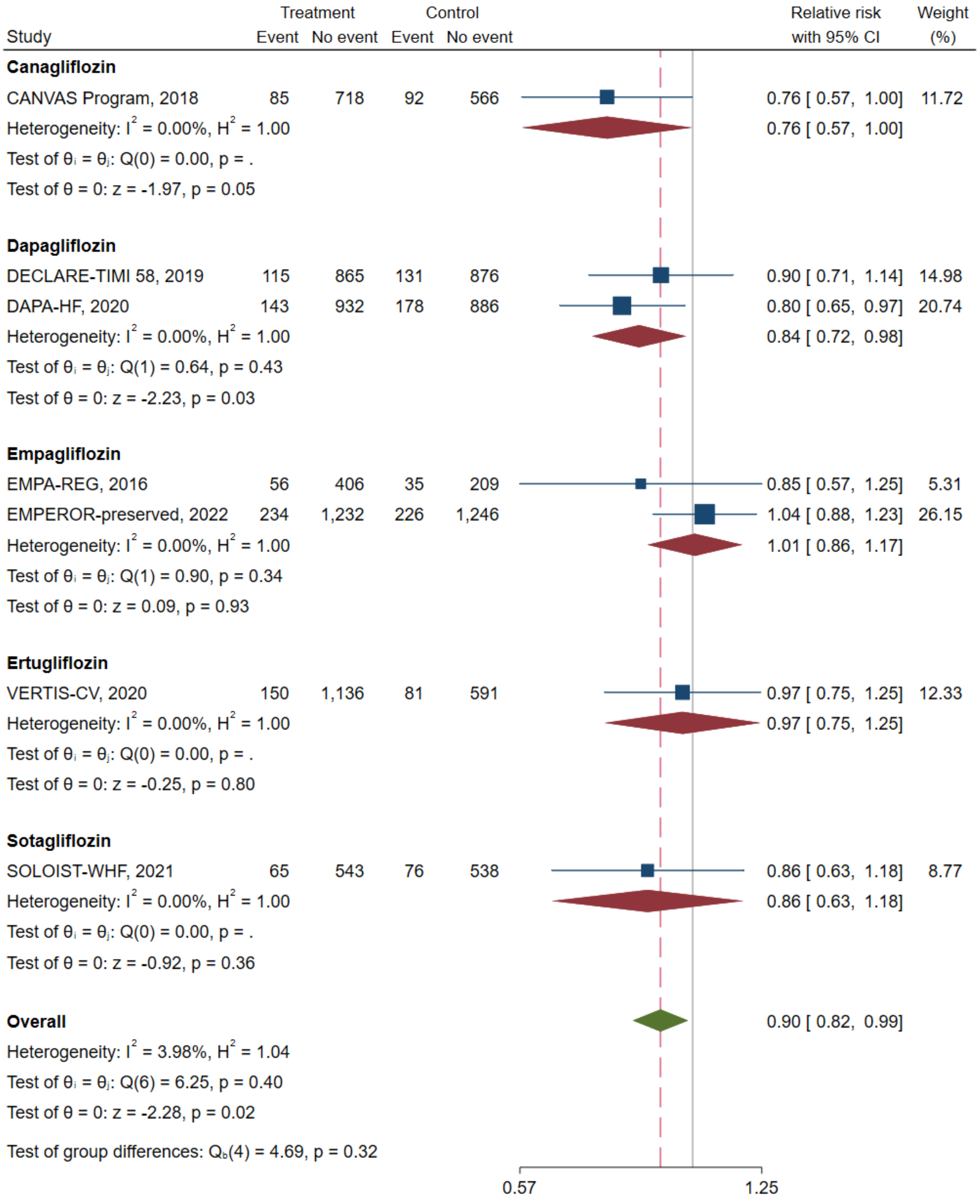

Supplementary Table S8: Confidence in Network Meta-Analysis of individual cardiovascular outcome

- **Composite cardiovascular death or heart failure hospitalization**

Comparison	Number of studies	Within-study bias	Reporting bias	Indirectness	Imprecision	Heterogeneity	Incoherence	Confidence rating	Reason(s) for downgrading
Canagliflozin:SoC	1	Some concerns	Some concerns	No concerns	No concerns	No concerns	Major concerns	Very low	Within-study bias, reporting bias, incoherence
Dapagliflozin:SoC	3	No concerns	Low risk	No concerns	No concerns	No concerns	Major concerns	Very low	Incoherence
Empagliflozin:SoC	2	No concerns	Low risk	No concerns	No concerns	Some concerns	Major concerns	Very low	Incoherence, Heterogeneity
Ertugliflozin:SoC	1	Some concerns	Low risk	No concerns	Some concerns	Some concerns	Major concerns	Very low	Within-study bias, Imprecision Incoherence, Heterogeneity
SoC:Sotagliflozin	2	No concerns	Low risk	No concerns	No concerns	No concerns	Major concerns	Very low	Incoherence
Canagliflozin:Dapagliflozin	0	Some concerns	Some concerns	No concerns	No concerns	Major concerns	Major concerns	Very low	Within-study bias, Reporting bias, Incoherence, Heterogeneity
Canagliflozin:Empagliflozin	0	Some concerns	Some concerns	No concerns	Some concerns	Some concerns	Major concerns	Very low	Within-study bias, Reporting bias, Incoherence, Imprecision, Heterogeneity
Canagliflozin:Ertugliflozin	0	Some concerns	Some concerns	No concerns	Some concerns	Some concerns	Major concerns	Very low	Within-study bias, Reporting bias, Incoherence, Imprecision, Heterogeneity
Canagliflozin:Sotagliflozin	0	Some concerns	Some concerns	No concerns	Some concerns	Some concerns	Major concerns	Very low	Within-study bias, Reporting bias, Incoherence, Imprecision, Heterogeneity

Dapagliflozin:Empagliflozin	0	No concerns	Low risk	No concerns	Major concerns	No concerns	Major concerns	Very low	Imprecision, Incoherence
Dapagliflozin:Ertugliflozin	0	Some concerns	Low risk	No concerns	Major concerns	No concerns	Major concerns	Very low	Within-study bias, Imprecision, Incoherence
Dapagliflozin:Sotagliflozin	0	No concerns	Low risk	No concerns	Some concerns	No concerns	Major concerns	Very low	Imprecision, Incoherence
Empagliflozin:Ertugliflozin	0	Some concerns	Low risk	No concerns	Major concerns	No concerns	Major concerns	Very low	Within-study bias, Imprecision, Incoherence
Empagliflozin:Sotagliflozin	0	No concerns	Low risk	No concerns	Some concerns	Some concerns	Major concerns	Very low	Imprecision, Incoherence, Heterogeneity
Ertugliflozin:Sotagliflozin	0	Some concerns	Low risk	No concerns	Some concerns	Some concerns	Major concerns	Very low	Within-study bias, Imprecision, Incoherence, Heterogeneity

- **Heart failure hospitalization**

Comparison	Number of studies	Within-study bias	Reporting bias	Indirectness	Imprecision	Heterogeneity	Incoherence	Confidence rating	Reason(s) for downgrading
Canagliflozin:SoC	2	Some concerns	Some concerns	No concerns	No concerns	Some concerns	Major concerns	Very low	Within-study bias, Reporting bias, Incoherence, Heterogeneity
Dapagliflozin:SoC	2	No concerns	Low risk	No concerns	No concerns	Some concerns	Major concerns	Very low	Incoherence, Heterogeneity
Empagliflozin:SoC	2	No concerns	Low risk	No concerns	No concerns	Some concerns	Major concerns	Very low	Incoherence, Heterogeneity
Empagliflozin:Soc	1	No concerns	Low risk	No concerns	No concerns	No concerns	Major concerns	Very low	Incoherence
Soc:Sotagliflozin	1	No concerns	Low risk	No concerns	No concerns	No concerns	Major concerns	Very low	Incoherence
Canagliflozin:Dapagliflozin	0	Some concerns	Some concerns	No concerns	No concerns	Major concerns	Major concerns	Very low	Within-study bias, Reporting bias, Incoherence, Heterogeneity

Canagliflozin:Empagliflozin	0	Some concerns	Some concerns	No concerns	No concerns	Major concerns	Major concerns	Very low	Within-study bias, Reporting bias, Incoherence, Heterogeneity
Canagliflozin:Soc	0	No concerns	Some concerns	No concerns	No concerns	Some concerns	Major concerns	Very low	Reporting bias, Incoherence, Heterogeneity
Canagliflozin:Sotagliflozin	0	No concerns	Some concerns	No concerns	Some concerns	Some concerns	Major concerns	Very low	Reporting bias, Imprecision, Incoherence, Heterogeneity
Dapagliflozin:Empagliflozin	0	No concerns	Low risk	No concerns	Some concerns	Some concerns	Major concerns	Very low	Incoherence, Imprecision, Heterogeneity
Dapagliflozin:Soc	0	No concerns	Low risk	No concerns	No concerns	Some concerns	Major concerns	Very low	Incoherence, Heterogeneity
Dapagliflozin:Sotagliflozin	0	No concerns	Low risk	No concerns	Major concerns	No concerns	Major concerns	Very low	Imprecision, Incoherence
Empagliflozin:Sotagliflozin	0	No concerns	Low risk	No concerns	Some concerns	Some concerns	Major concerns	Very low	Imprecision, Incoherence, Heterogeneity
Soc:SoC	0	No concerns	Low risk	No concerns	Some concerns	Some concerns	Major concerns	Very low	Imprecision, Incoherence, Heterogeneity
SoC:Sotagliflozin	0	No concerns	Low risk	No concerns	Some concerns	Some concerns	Major concerns	Very low	Imprecision, Incoherence, Heterogeneity

- **Cardiovascular death**

Comparison	Number of studies	Within-study bias	Reporting bias	Indirectness	Imprecision	Heterogeneity	Incoherence	Confidence rating	Reason(s) for downgrading
Canagliflozin:SoC	2	Some concerns	Some concerns	No concerns	Some concerns	Some concerns	Major concerns	Very low	Within-study bias, Reporting bias, Incoherence, Heterogeneity, Imprecision
Dapagliflozin:SoC	2	No concerns	Low risk	No concerns	Some concerns	Some concerns	Major concerns	Very low	Imprecision, Incoherence, Heterogeneity
Empagliflozin:SoC	2	No concerns	Low risk	No concerns	Some concerns	Some concerns	Major concerns	Very low	Imprecision, Incoherence, Heterogeneity
Empagliflozin:Soc	1	No concerns	Low risk	No concerns	Some concerns	Some concerns	Major concerns	Very low	Imprecision, Incoherence, Heterogeneity
Ertugliflozin:SoC	1	Some concerns	Low risk	No concerns	Major concerns	No concerns	Major concerns	Very low	Within-study bias, Imprecision, Incoherence
SoC:Sotagliflozin	1	No concerns	Low risk	No concerns	Major concerns	No concerns	Major concerns	Very low	Imprecision, Incoherence
Canagliflozin:Dapagliflozin	0	Some concerns	Some concerns	No concerns	Major concerns	No concerns	Major concerns	Very low	Within-study bias, Reporting bias, Incoherence, Imprecision
Canagliflozin:Empagliflozin	0	Some concerns	Some concerns	No concerns	Some concerns	Some concerns	Major concerns	Very low	Within-study bias, Reporting bias, Incoherence, Heterogeneity, Imprecision
Canagliflozin:Ertugliflozin	0	Some concerns	Some concerns	No concerns	Major concerns	No concerns	Major concerns	Very low	Within-study bias, Reporting bias, Imprecision, Incoherence
Canagliflozin:Soc	0	No concerns	Some concerns	No concerns	Some concerns	Some concerns	Major concerns	Very low	Reporting bias, Incoherence, Heterogeneity, Imprecision
Canagliflozin:Sotagliflozin	0	Some concerns	Some concerns	No concerns	Major concerns	No concerns	Major concerns	Very low	Within-study bias, Reporting bias, Imprecision, Incoherence

Dapagliflozin:Empagliflozin	0	No concerns	Low risk	No concerns	Major concerns	No concerns	Major concerns	Very low	Imprecision, Incoherence
Dapagliflozin:Ertugliflozin	0	Some concerns	Low risk	No concerns	Major concerns	No concerns	Major concerns	Very low	Within-study bias, Imprecision, Incoherence
Dapagliflozin:Soc	0	No concerns	Low risk	No concerns	Major concerns	No concerns	Major concerns	Very low	Imprecision, Incoherence
Dapagliflozin:Sotagliflozin	0	No concerns	Low risk	No concerns	Major concerns	No concerns	Major concerns	Very low	Imprecision, Incoherence
Empagliflozin:Ertugliflozin	0	Some concerns	Low risk	No concerns	Major concerns	No concerns	Major concerns	Very low	Within-study bias, Imprecision, Incoherence
Empagliflozin:Sotagliflozin	0	No concerns	Low risk	No concerns	Major concerns	No concerns	Major concerns	Very low	Imprecision, Incoherence
Ertugliflozin:Soc	0	No concerns	Low risk	No concerns	Major concerns	No concerns	Major concerns	Very low	Imprecision, Incoherence
Ertugliflozin:Sotagliflozin	0	Some concerns	Low risk	No concerns	Major concerns	No concerns	Major concerns	Very low	Within-study bias, Imprecision, Incoherence
Soc:SoC	0	No concerns	Low risk	No concerns	Major concerns	No concerns	Major concerns	Very low	Imprecision, Incoherence
Soc:Sotagliflozin	0	No concerns	Low risk	No concerns	Major concerns	No concerns	Major concerns	Very low	Imprecision, Incoherence

- **All-cause mortality**

Comparison	Number of studies	Within-study bias	Reporting bias	Indirectness	Imprecision	Heterogeneity	Incoherence	Confidence rating	Reason(s) for downgrading
Canagliflozin:SoC	2	Some concerns	Some concerns	No concerns	No concerns	Major concerns	Major concerns	Very low	Within-study bias, Reporting bias, Incoherence, Heterogeneity
Dapagliflozin:SoC	2	No concerns	Low risk	No concerns	No concerns	Major concerns	Major concerns	Very low	Incoherence, Heterogeneity
Empagliflozin:SoC	2	No concerns	Low risk	No concerns	Major concerns	No concerns	Major concerns	Very low	Imprecision, Incoherence
Ertugliflozin:SoC	1	Some concerns	Low risk	No concerns	Major concerns	No concerns	Major concerns	Very low	Within-study bias, Imprecision, Incoherence
SoC:Sotagliflozin	1	No concerns	Low risk	No concerns	Major concerns	No concerns	Major concerns	Very low	Imprecision, Incoherence
Canagliflozin:Dapagliflozin	0	Some concerns	Some concerns	No concerns	Major concerns	No concerns	Major concerns	Very low	Within-study bias, Reporting bias, Incoherence, Imprecision
Canagliflozin:Empagliflozin	0	Some concerns	Some concerns	No concerns	Some concerns	Some concerns	Major concerns	Very low	Within-study bias, Reporting bias, Incoherence, Heterogeneity, Imprecision
Canagliflozin:Ertugliflozin	0	Some concerns	Some concerns	No concerns	Major concerns	No concerns	Major concerns	Very low	Within-study bias, Reporting bias, Incoherence, Imprecision
Canagliflozin:Sotagliflozin	0	Some concerns	Some concerns	No concerns	Major concerns	No concerns	Major concerns	Very low	Within-study bias, Reporting bias, Incoherence, Imprecision
Dapagliflozin:Empagliflozin	0	No concerns	Low risk	No concerns	Some concerns	Some concerns	Major concerns	Very low	Incoherence, Imprecision, Heterogeneity
Dapagliflozin:Ertugliflozin	0	Some concerns	Low risk	No concerns	Major concerns	No concerns	Major concerns	Very low	Within-study bias, Imprecision, Incoherence
Dapagliflozin:Sotagliflozin	0	No concerns	Low risk	No concerns	Major concerns	No concerns	Major concerns	Very low	Imprecision, Incoherence

Empagliflozin:Ertugliflozin	0	Some concerns	Low risk	No concerns	Major concerns	No concerns	Major concerns	Very low	Within-study bias, Imprecision, Incoherence
Empagliflozin:Sotagliflozin	0	No concerns	Low risk	No concerns	Major concerns	No concerns	Major concerns	Very low	Imprecision, Incoherence
Ertugliflozin:Sotagliflozin	0	Some concerns	Low risk	No concerns	Major concerns	No concerns	Major concerns	Very low	Within-study bias, Imprecision, Incoherence
Canagliflozin:Dapagliflozin	0	Some concerns	Some concerns	No concerns	Major concerns	No concerns	Major concerns	Very low	Within-study bias, Reporting bias, Incoherence, Imprecision

- **Serious adverse events**

Comparison	Number of studies	Within-study bias	Reporting bias	Indirectness	Imprecision	Heterogeneity	Incoherence	Confidence rating	Reason(s) for downgrading
Canagliflozin:SoC	1	Some concerns	Some concerns	No concerns	No concerns	No concerns	Major concerns	Very low	Within-study bias, Reporting bias, Incoherence
Dapagliflozin:SoC	2	No concerns	Low risk	No concerns	No concerns	Some concerns	Major concerns	Very low	Heterogeneity, Incoherence
Empagliflozin:SoC	3	No concerns	Low risk	No concerns	No concerns	Some concerns	Major concerns	Very low	Heterogeneity, Incoherence
SoC:Sotagliflozin	1	No concerns	Low risk	No concerns	No concerns	Major concerns	Major concerns	Very low	Heterogeneity, Incoherence
Canagliflozin:Dapagliflozin	0	Some concerns	Some concerns	No concerns	No concerns	Some concerns	Major concerns	Very low	Within-study bias, Reporting bias, Incoherence, Heterogeneity
Canagliflozin:Empagliflozin	0	Some concerns	Some concerns	No concerns	No concerns	Some concerns	Major concerns	Very low	Within-study bias, Reporting bias, Heterogeneity, Incoherence
Canagliflozin:Sotagliflozin	0	Some concerns	Some concerns	No concerns	No concerns	Some concerns	Major concerns	Very low	Within-study bias, Reporting bias, Heterogeneity, Incoherence
Dapagliflozin:Empagliflozin	0	No concerns	Low risk	No concerns	No concerns	Some concerns	Major concerns	Very low	Heterogeneity, Incoherence
Dapagliflozin:Sotagliflozin	0	No concerns	Low risk	No concerns	Some concerns	Some concerns	Major concerns	Very low	Heterogeneity, Incoherence, Imprecision
Empagliflozin:Sotagliflozin	0	No concerns	Low risk	No concerns	No concerns	Major concerns	Major concerns	Very low	Heterogeneity, Incoherence

- **Any adverse events**

Comparison	Number of studies	Within-study bias	Reporting bias	Indirectness	Imprecision	Heterogeneity	Incoherence	Confidence rating	Reason(s) for downgrading
Canagliflozin:SoC	2	Some concerns	Some concerns	No concerns	Some concerns	Some concerns	Major concerns	Very low	Within-study bias, Reporting bias, Incoherence, Heterogeneity, Imprecision
Dapagliflozin:SoC	2	Some concerns	Low risk	No concerns	No concerns	Some concerns	Major concerns	Very low	Within-study bias, Incoherence, Heterogeneity
Empagliflozin:SoC	3	No concerns	Low risk	No concerns	No concerns	No concerns	Major concerns	Very low	Incoherence
SoC:Sotagliflozin	1	No concerns	Low risk	No concerns	No concerns	Some concerns	Major concerns	Very low	Incoherence, Heterogeneity
Canagliflozin:Dapagliflozin	0	Some concerns	Some concerns	No concerns	Some concerns	Some concerns	Major concerns	Very low	Within-study bias, Reporting bias, Incoherence, Heterogeneity, Imprecision
Canagliflozin:Empagliflozin	0	Some concerns	Some concerns	No concerns	Some concerns	Some concerns	Major concerns	Very low	Within-study bias, Reporting bias, Incoherence, Heterogeneity, Imprecision
Canagliflozin:Sotagliflozin	0	Some concerns	Some concerns	No concerns	Some concerns	Some concerns	Major concerns	Very low	Within-study bias, Reporting bias, Incoherence, Heterogeneity, Imprecision
Dapagliflozin:Empagliflozin	0	Some concerns	Low risk	No concerns	No concerns	Some concerns	Major concerns	Very low	Within-study bias, Incoherence, Heterogeneity
Dapagliflozin:Sotagliflozin	0	No concerns	Low risk	No concerns	No concerns	Major concerns	Major concerns	Very low	Incoherence, Heterogeneity
Empagliflozin:Sotagliflozin	0	No concerns	Low risk	No concerns	No concerns	Some concerns	Major concerns	Very low	Incoherence, Heterogeneity
